# Identification of functional pathways and hub genes associated with the heterochronic development of sugarcane axillary buds and sett roots through multi-omics analysis

**DOI:** 10.3389/fpls.2025.1551783

**Published:** 2025-03-14

**Authors:** Jiabao Zheng, Xiaoyu Huang, Yanli Wei, Wenyan Li, Baoshan Chen, Wenlan Li

**Affiliations:** ^1^ State Key Laboratory for Conservation and Utilization of Subtropical Agro-bioresources, College of Life Science and Technology, Guangxi University, Nanning, China; ^2^ Guangxi Key Laboratory of Sugarcane Biology, College of Agriculture, Guangxi University, Nanning, China

**Keywords:** sugarcane, sprouting and rooting strategy, heterochrony, metabolomics analysis, transcriptomics analysis, WGCNA

## Abstract

**Introduction:**

Sugarcane is primarily propagated for large-scale agricultural production through vegetative reproduction by planting stem cuttings. Development of sprout and sett root from the cuttings is essential for sugarcane plant to adapt to the field environment. We observed asynchronous development during the sprouting of stem cuttings in two sibling sugarcane cultivars sharing the same parent in cross breeding: the axillary buds of cultivar ZZ2 (ZZ2B) sprout earlier, while the sett roots of ZZ9 (ZZ9R) emerge sooner.

**Methods:**

Comparison of the sett root architecture, soluble sugar content, plant hormone levels and gene expression profiles during sprouting.

**Results:**

We found that ZZ9 has a lower root cortex thickness ratio and a higher vascular cylinder thickness ratio. We also identified significant differences in the levels of soluble sugars, 3-Indolebutyric acid (IBA), N6-isopentenyladenosine (IPA), cis-Zeatin (cZ), Abscisic Acid (ABA), Gibberellin A3 (GA_3_), Gibberellin A7 (GA_7_), (±)-Jasmonic acid (JA), and N-((-)-jasmonoyl)-Sisoleucine (JA-Ile) between these cultivars. cuttings. In addition, we identified differentially expressed genes through transcriptomic analysis and discovered, via GO and KEGG enrichment analyses, that negative regulation of external stimulus response is a key to the preference of ZZ2 for early bud sprouting. The Twin-arginine translocation complex (Tat) significantly influences the preference of ZZ9’s root emergence. Furthermore, weighted gene co-expression network analysis (WGCNA) revealed that specific metabolic processes in seed coat mucilage uniquely determine the asynchronous development of sett roots and axillary buds.

**Discussion:**

These findings provide a theoretical foundation and new perspective for understand asynchronous development in sugarcane production, offering novel insights for breeding high-quality varieties.

## 1 Introduction

Sugarcane (*Saccharum hybrid cultivar*) is a widely cultivated economic crop globally and is primarily used to produce industrial raw materials, such as sugar and ethanol (Waclawovsky et al., 2010). Sugar extracted from sugarcane accounts for 80% of global sugar production (Ali et al., 2019), while ethanol produced from sucrose contributes to 40% of global ethanol production (Boaretto et al., 2021). Sugarcane bagasse can also be utilized to produce industrial products, including biofuels, bioelectricity, bioplastics, bioadsorbents, and organic acids, through various processes such as pyrolysis, liquefaction, gasification, cogeneration, lignin conversion, and cellulose fermentation (Shabbirahmed et al., 2022). Consequently, sugarcane possesses substantial economic value and industrial importance, which underscores the need to develop and explore high-yield, stress-resistant cultivars.

Considering that sexual reproduction in sugarcane diverts nutrients to meet the energy demands of reproduction and development and the complex genetic background of the allopolyploid nature of sugarcane leads to unstable traits in offspring, agricultural production of sugarcane primarily relies on clonal propagation through stem cuttings (Dinesh Babu et al., 2022). The axillary buds and sett roots of sugarcane stem cuttings exhibit differences in sprouting timing, which demonstrates heterochronic development. Heterochronic development refers to temporal differences in the developmental processes of various parts of an organism. This phenomenon can manifest as differences in the initiation, rate, or cessation of development among different tissues or organs (Klingenberg, 1998). In plants, heterochronic development of roots and buds is typically driven by the timing regulation of hormones, heterochronic gene expression, and adaptation to environmental conditions (Buendía-Monreal and Gillmor, 2018). Heterochronic development plays a crucial role in plant survival and adaptability, by ensuring that plants can prioritize the development of essential structures in resource-limited environments, which maximizes their chances of survival (Geuten and Coenen, 2013). However, no reports are currently available on the specific effects of heterochronic development in sugarcane stem cuttings on its seedling stage, tillering stage, elongation stage, and maturity stage. Only empirical observations from sugarcane producers exist. This gap has motivated us to conduct a preliminary study on the heterochronic development of sugarcane stem cuttings.

The root system of sugarcane is fibrous and serves as a vital organ for water and nutrient absorption. It also senses changes in the soil and other environmental factors to regulate its own growth and development. The sett roots of sugarcane emerge from nodal root points on the stem and are also known as temporary or adventitious roots. In their early stages, these roots do not branch; axillary roots begin to from only after reaching a length of approximately 5–10 cm. The morphological structure of sett roots is relatively delicate, with limited capacity for nutrient and water absorption. Without additional intervention, these roots undergo programmed senescence and apoptosis within a development period of 4–6 weeks. During this period, sett roots are gradually replaced by newly emerging roots from the sugarcane seedling, rather than the stem cutting. These new roots are referred to as shoot roots (Smith et al., 2005).

The water and nutrients stored within the sugarcane stem cuttings provide essential resources for the initial development of the seedlings, which supports the growth of axillary buds into seedlings and the sprouting of sett roots. As the sett roots complete their initial role of absorbing water and nutrients from the environment and undergo physiological and programmed senescence, the primary source of growth resources in sugarcane shifts to the shoot roots developed from the seedlings. This transition signifies the complete shift of sugarcane growth from a heterotrophic to an autotrophic state (Ball-Coelho et al., 1992). The period following sprouting is the most critical phase in the life of green plants, as the developing seedlings must attain an autotrophic state; otherwise, the nutrients stored in the seed or stem cuttings will be depleted (Kircher and Schopfer, 2012). Earlier sprouting of the sett roots sprout results in faster develop and longer persistence, which enable them to absorb water and nutrients from the environment more effectively. This phenomenon supports the growth and development of the sugarcane seedlings (Cunha et al., 2020). This strategy of root emergence preceding shoot growth enhances the efficiency of sugarcane sprouting and seedling development, particularly under extreme conditions such as water scarcity (de Abreu et al., 2021). Studies show that significant anatomical differences exist in the root systems of various sugarcane varieties, which are closely associated with their variations in root hydraulic conductance (water uptake capacity). Sugarcane varieties with a larger xylem area in their root systems generally exhibit higher root hydraulic conductance. Drought-resistant varieties, such as H69-8235, have a greater xylem area, which enhances their water absorption capacity. Roots with a larger stele area demonstrate higher hydraulic conductance given that the stele serves as the primary pathway for upward water transport through the root system. The root system may have a denser conduit network or fewer air spaces (or embolisms), and these structural differences affect the effective absorption and transport of water. Overall, the anatomical structure of the sugarcane root system, particularly the size of the xylem and stele, as well as the structure of the conduits, directly influences its water absorption capacity and hydraulic conductance. Therefore, these anatomical differences among varieties may be a fundamental cause of the variation in water absorption capacity (Saliendra and Meinzer, 1992).

When the sugarcane stem nodes are excised and placed under suitable conditions, the stored substances inside gradually undergo hydrolysis. This process provides the necessary energy and proteins required for the sprouting of axillary buds and sett roots. The respiration of various tissues is enhanced, enzyme activities increase, and the conversion of stored nutrients into small molecules is promoted, thus breaking the dormancy state. The composition of stored sugars in seeds varies and can be categorized based on water solubility into soluble sugars (such as glucose, fructose, sucrose, maltose, stachyose, and raffinose) and insoluble carbohydrates, primarily starch. During sprouting, starch reserves are mobilized and hydrolyzed into soluble sugars, which subsequently undergo metabolic processing to supply energy for seedling establishment. Sucrose plays a dual role in maintaining cellular osmotic balance and facilitating seed imbibition. In starchy seeds, sucrose predominates among soluble sugars during the initial phase of sprouting, whereas glucose serves as a pivotal component throughout sugar metabolism, with its concentration positively correlated with sprouting efficiency (Pastorini et al., 2003; Sánchez-Linares et al., 2012). As a fundamental molecular component in biological processes, soluble proteins play a crucial role in seed germination and are intricately linked to various life activities. During plant growth and development, proteins not only serve as an essential nitrogen source and energy provider for seed germination but also function as key regulators of the germination process. Studies have shown that fluctuations in soluble protein content during germination can, to some extent, serve as an indicator of the intensity of physiological and metabolic activities, thereby offering valuable insights into the progression of seed germination (Tada and Kashimura, 2009; Zhang et al., 2017). Currently, there is a lack of systematic research on the role of soluble compounds in sugarcane seed stalks during sprouting and root development. Therefore, the dynamic changes in soluble compounds observed during seed germination may serve as a reference indicator for studying sugarcane sprouting and root formation.

The sprouting timing of axillary buds and sett roots in sugarcane is largely the result of the regulatory balance between various plant hormones, including auxins (Aux), cytokinins (CKs), gibberellins (GAs), abscisic acid (ABAs), 1-aminocyclopropane-1-carboxylic acid (preETH), salicylic acid (SA), jasmonates (JAs), and brassinosteroids (BRs). Plant hormones are terminal products of gene expression, transcription, and various physiological regulatory processes. The use of UHPLC–MS/MS technology enables rapid, sensitive, and high-throughput detection of plant hormones (Floková et al., 2014). Using partial least squares discriminant analysis (PLS-DA) effectively reveals the impact and role of key differential hormones on biological phenotypes (Wang et al., 2022).

Transcriptome analysis can compensate for the blind spots of metabolome analysis at the whole-gene transcription level, which explains the changes in plant hormone levels from the perspective of gene expression regulation. Although single-omics data analysis can explain parts of the genetic information and metabolic pathways of a species, it struggles to capture complex biological processes and regulatory networks. Integrating multi-omics data can compensate for data gaps and reduce noise in single-omics analyses. Different levels of data can also validate each other, which reduces false positives. More importantly, multi-omics data integration facilitates the study of phenotypes and regulatory mechanisms in biological models. Joint analysis of transcriptomics and metabolomics is a common multi-omics approach, which enables the construction of gene and plant hormone interaction networks. This approach more comprehensively and effectively explains and reveals the core mechanisms behind the biological phenomena and questions under investigation (Meng et al., 2022; Shen et al., 2022; Xing et al., 2022).

The modern sugarcane cultivar is a complex polyploidy produced by interspecific crosse of *S.* sp*ontaneum* (with high abiotic stress tolerance and ratooning capacity) and *S. officinarum* (with high sugar content), followed by multiple backcrosses with *S. officinarum* (Wang et al., 2024).

Recently, Muqing et al. sequenced the hybrid sugarcane cultivar ZZ1 using a combination of Illumina, PacBio HiFi, and Hi-C sequencing technologies. The final assembled genome size was 10.4 Gb, with 87% (9.1/10.4 Gb) of the sequences anchored to 114 pseudochromosomes, representing a significant improvement compared to previously published, partially assembled genomes of cultivated sugarcane. The BUSCO assessment indicated a high level of genome completeness, and the LAI (LTR Assembly Index) value was calculated to be 12.27, suggesting that this assembly can serve as a high-quality reference genome (Bao et al., 2024).

ZZ1 shares the same parental lineage (ROC25 and YZ89-7) with ZZ2 and ZZ9, resulting in an identical genetic background. Therefore, using ZZ1 as the reference genome in this study is highly appropriate. ZZ2 is a thin-stemmed sugarcane variety with low sugar content and weak stress tolerance, while ZZ9 is a thick-stemmed variety with high stress tolerance and high sugar content. The two varieties display a marked contrast in traits. In large-scale screening for high-quality sugarcane germplasm, we unexpectedly discovered that the two sugarcane varieties exhibit asynchronous development in their stem cuttings: ZZ2 exhibits faster sprouting of axillary buds, while ZZ9 shows faster emergence of sett roots. The two varieties thus serve as excellent materials for studying the asynchronous development of sprouting and rooting in sugarcane stem cuttings.

This study provides supplementary observations and data on sugarcane setts during the critical periods from dormancy to bud emergence. In addition, by focusing on two cultivars that exhibit contrasting phenotypes during the sprouting phase, we explore how sprouting and root initiation affect the subsequent growth and development of sugarcane. These insights are valuable for the large-scale agricultural production of sugarcane, which primarily relies on vegetative propagation.

## 2 Materials and methods

### 2.1 Plant materials and growth conditions

ZZ2 and ZZ9 originate from the sugarcane experimental base at the Agricultural Science New Campus of Guangxi University.

The selection criteria for sugarcane stem nodes were as follows: the first node was defined as the thickened region exposed at the top of the sugarcane stem (**Supplementary Figure S3**). For each cane, the 10th to the 20th nodes were selected for cultivation. These nodes were placed in plant culture boxes and continuously incubated for 12 days without direct contact with water. The culture conditions were as follows: air humidity maintained at 75%, temperature at 27°C, and the environment was kept dark (XUNON AS-R1760, China)

Sugarcane axillary buds and sett roots were collected at days 0, 1, 2, 3, and 4, with three biological replicates per time point, resulting in a total of 60 samples for UHPLC-MS/MS and RNA-seq analyses.

For axillary buds, the sampling target includes dormant or sprouting buds within the bud eyes.

For sett roots, the sampling target includes the root primordium region, which contains the sett roots.

Each biological replicate consists of samples from 20 stem nodes.

On day 12, root tissue samples were collected from each sett roots at a point 3 cm from the root tip. Fifteen biological replicates were prepared for each variety, and the samples were placed in clean 50 ml centrifuge tubes. An appropriate amount of FAA fixative (Formaldehyde-Acetic Acid-Ethanol, 50%, Biosharp, China) was added to completely submerge the material. The samples were left to fix at 4°C for 24 h for subsequent root paraffin sectioning.

B = axillary buds, R =sett roots.

### 2.2 Preparation and observation of root paraffin sections

The fully fixed materials of ZZ2R and ZZ9R were transferred into 50 ml centrifuge tubes. The first gradient dehydration was performed using ethanol solutions (Sangon Biotech, China) with concentrations of 50%, 60%, 70%, 80%, and 90%, followed by two dehydrations with absolute ethanol. The sugarcane root tissues were cleared using TO clearing agents (Tissue-Clearing Reagent iTOMEI-D [for Plants], Aladdin, China) at concentrations of 30%, 50%, 70%, and 100% (solvent: absolute ethanol). Each step requires vacuum treatment for 20 min followed by standing for 10 min before proceeding to the next concentration. After the clearing process, paraffin (52°C -54°C, Paraffin with Ceresin, MP 52°C -54°C, BBI, China) was added to the samples, which were incubated in a 55°C oven for 4–5 weeks, with fresh paraffin added as needed. The sugarcane root tissues were embedded using a paraffin embedding machine (LEICA EG1150, China), and then sectioned with an automatic microtome (LEICA RM2255, China) to a thickness of 8–12 μm. The sections were placed on a drying platform after cutting. Paraffin was sequentially removed from the sugarcane root tissues by treating them with TO solutions at concentrations of 100%, 70%, 50%, and 30% (solvent: absolute ethanol), followed by absolute ethanol, and then ethanol solutions at concentrations of 90%, 80%, 70%, and 50%. Each treatment step lasted for 10 min. Next, the tissues were stained with hematoxylin (BBI, China) for 10 min, and then rinsed with pure water. The tissues were dehydrated using a series of ethanol solutions at concentrations of 50%, 70%, 80%, and 90%, followed by absolute ethanol, and then TO clearing agents at concentrations of 30%, 50%, 70%, and 100% (solvent: absolute ethanol). Finally, the sections were mounted with neutral balsam mounting medium (BBI, China) and observed under an optical microscope (Olympus BX63, Japan) for imaging.

### 2.3 Detection of soluble sugars and soluble proteins

Sucrose standard solutions with concentrations ranging from 0 μg/mL to 100 μg/mL were prepared. Six 18×180 mm glass test tubes were collected, and 0.5 mL of anthrone (ethyl acetate) reagent was added to each tube. A total of 5 mL of concentrated sulfuric acid, was slowly added, the test tubes were sealed, and the mixture was mixed well. The stoppers were removed, and the tubes were heated in a boiling water bath for 60 s. The tubes were allowed to cool Before the solution was transferred to a 10 mL glass vial and mixed thoroughly. A 200 μL aliquot of the solution was collected and placed in a microplate. A microplate reader was used to measure the absorbance at UV_630_ and the standard curve for soluble sugars was plotted.

The experimental material was ground into powder using liquid nitrogen. Then, 0.1 g of the powder was weighed and added to 5 mL of ddH_2_O to form a mixture. The mixture was centrifuged at 12,000 g for 3 min. Next, 0.5 mL of the supernatant was collected and added to 0.5 mL of anthrone solution (BBI, China) (solvent: ethyl acetate (Sangon Biotech, China)) along with 1.5 mL of ddH_2_O. Thereafter, 5 mL of concentrated sulfuric acid (Aladdin, China) was added. The mixture was thoroughly shaken, and then heated in a boiling water bath for 60 s. A microplate reader (Synergy H1, BioTek Instruments, Inc., United States) was used to measure and record the absorbance at UV_630_.

Protein standard solutions with concentrations ranging from 0% to 100% were prepared. Seven glass test tubes were collected, and the protein standard solutions of varying concentrations along with water were added to each tube. Then, 5 mL of G-250 solution was added, mixed thoroughly, and left to stand for 5–20 min. A 200 μL aliquot of each solution were collected and placed in a microplate. A microplate reader was used to measure the absorbance at UV_595_ and the standard curve for soluble proteins was plotted.

A total of 0.1 g of the powder was weighed and added to 5 mL of PBS solution (50 mmol/L, pH 7.8, 4°C) containing 1% PVP K-30 (BBI, China) to form a mixture. The mixture was centrifuged at 12,000 g for 3 min, and the supernatant was collected as the crude enzyme extract. Then, 0.1 mL of the crude enzyme extract sample was collected and added to 5 mL of Coomassie Brilliant Blue G-250 solution (BBI, China). The mixture was thoroughly shaken, allowed to stand for 5 min, and the absorbance was measured and recorded at UV_595_ using a microplate reader.

The contents of soluble sugars and soluble proteins were calculated based on the standard curve.

### 2.4 Metabolomics detection and differential hormone data analysis

This project employs a targeted metabolomics approach using UHPLC–MS/MS (Šimura et al., 2018) to detect 24 types of endogenous plant hormones across 60 experimental samples, with three biological replicates set for each sample. The detection data include eight categories of plant hormones: Auxs (Methyl 3-indolylacetate, Indole-3-acetic acid, 3-Indolebutyric acid, Indole-3-carboxaldehyde), CKs (trans-Zeatin-riboside, trans-Zeatin, cis-Zeatin, DL-Dihydrozeatin, N6-isopentenyladenosine, N6-(delta 2-Isopentenyl)-adenine, Kinetin), GAs (Gibberellin A1, Gibberellin A3, Gibberellin A4, Gibberellin A7), ABA, preETH (1-Aminocyclopropanecarboxylic acid), SA (Methyl salicylate, Salicylic acid), Jas (Methyl jasmonate, Dihydrojasmonic Acid, N-((-)-jasmonoyl)-S-isoleucine, (±)-Jasmonic acid), and BRs (Brassinolide).

The samples were grinded into powder by liquid nitrogen. Then a 25 mg aliquot of each individual sample was precisely weighed and transferred to an Eppendorf tube. After the addition of 1,000 μL of extract solution (50% acetonitrile in water, precooled at −40°C, containing isotopically labeled internal standard mixture), the samples were vortexed for 30 s and sonicated for 10 min in the ice-water bath, and homogenized at 40 Hz for 4 min. The homogenate and sonicate circle was repeated twice. After centrifugation (10 min, 12,000 rpm, and 4°C), a 900 μL aliquot of the supernatant was further purified with SPE. The SPE cartridges were washed with 1 mL of methanol and then equilibrated with 1 mL 50% ACN/H_2_O (v/v). After loading samples (supernatant obtained following the procedure described above), the flow-through fraction was discarded. The sample for the UHPLC was collected by washing the column with 1 mL of 60% ACN/H_2_O (v/v). After this single-step SPE, the samples were evaporated to dryness under a gentle stream of nitrogen and were reconstituted with 90 μL of 10% ACN/H_2_O (v/v). All the samples were vortexed for 30 s and sonicated for 5 min in the ice-water bath. After centrifugation (15 min, 12,000 rpm, and 4°C), the clear supernatant was subjected to UHPLC–MS/MS analysis.

Stock solutions were individually prepared by dissolving or diluting each standard substance to obtain a final concentration of 10 mmol/L. An aliquot of each of the stock solutions was transferred to a 10 mL flask to form a mixed working standard solution. Several calibration standard solutions were then prepared by stepwise dilution of this mixed standard solution (containing isotopically-labelled internal standard mixture in identical concentrations with the samples).

The UHPLC separation was conducted using an EXIONLC System (Sciex), which was equipped with a Waters ACQUITY UPLC CSH C18 column (150 × 2.1 mm, 1.7 μm, Waters). The mobile phase A was 0.01% formic acid in water, and the mobile phase B was 0.01% formic acid in acetonitrile. The column temperature was set at 50°C. The auto-sampler temperature was set at 4°C and the injection volume was 5 μL.

A SCIEX 6500 QTRAP+ triple quadrupole mass spectrometer (Sciex), which was equipped with an IonDrive Turbo V electrospray ionization (ESI) interface, was applied for assay development. Typical ion source parameters were as follows: curtain gas = 40 psi, ionspray voltage = ± 4500 V, temperature = 475°C, ion source gas 1 = 30 psi, and ion source gas 2 = 30 psi.

The MRM parameters for each of the targeted analytes were optimized using flow injection analysis, by injecting the standard solutions of the individual analytes, into the API source of the mass spectrometer. Several most sensitive transitions were used in the MRM scan mode to optimize the collision energy for each Q1/Q3 pair. Among the optimized MRM transitions per analyte, the Q1/Q3 pairs with the highest sensitivity and selectivity were selected as “quantifier” for quantitative monitoring. The additional transitions acted as “qualifier” for verifying the identity of the target analytes.

SCIEX Analyst Work Station Software (Version 1.6.3) and SCIEX OSQ were employed for MRM data acquisition and processing.

Calibration solutions were subjected to UPLC–MRM–MS/MS analysis using the methods described above. Least square method was used for regression fitting. 1/x weighting was applied in the curve fitting given that it provided highest accuracy and correlation coefficient (R2). The level was excluded from the calibration if the accuracy of calibration was not within the range of 80% to 120%.

The calibration standard solution was diluted stepwise, with a dilution factor of 2. These standard solutions were subjected to UHPLC–MRM–MS analysis. The signal-to-noise ratios (S/N) were used to determine the lower limits of detection (LLODs) and lower limits of quantitation (LLOQs). The LLODs and LLOQs were defined as the analyte concentrations that led to peaks with S/N of 3 and 10, respectively, according to the US FDA guideline for bioanalytical method validation.

The precision of the quantitation was measured as the relative standard deviation (RSD), which was determined by injecting analytical replicates of a QC sample. The accuracy of quantitation was measured as the analytical recovery of the QC sample determined. The percent recovery was calculated as [(mean observed concentration)/(spiked concentration)] × 100%.

The final concentration (CF, nmol/L) is obtained by calculating concentration (CC, nmol/L) multiplied by the dilution factor (Dil). The metabolite concentration (CM, nmol/kg) is calculated by multiplying the final concentration (CF, nmol/L) by the final volume (V, μL), and dividing by the mass (m, mg) of the sample. “N/A” indicates that the targeted metabolites were not detectable in the corresponding samples. The results highlighted in blue represent concentrations below the LLOQs, which suggests relatively poorer quantitative accuracy.


$$C_{M}[\text{nmol}\cdot kg^{-1}]=\frac{C_{F}[\text{nmol}\cdot L^{-1}]\cdot V[\mu L]}{m[mg]}$$

Preprocessed metabolomic data were organized into datasets corresponding to the shoot and root tissues of ZZ2 and ZZ9, according to cultivation time. Differential metabolites were screened using PLS-DA in SIMCA (v14.1). A permutation test with 999 random iterations was conducted to validate the robustness of the model, and metabolites with a VIP score ≥ 1 were designated as differential hormones for each time point. To ensure rigorous consideration of the regulatory biological roles of plant hormones, a threshold was set requiring that hormones exhibit significant differences at no fewer than three time points. This threshold served as the criterion for the final determination of hormones with differential content levels.

### 2.5 Total RNA extraction and transcriptome data analysis

Total RNA was extracted from the axillary buds and sett roots of ZZ2 and ZZ9 using the Trizol reagent method (TaKaRa, China). The RNA concentration was measured using a micro-spectrophotometer (Thermo Scientific NanoDrop 2000, United States), and RNA integrity was verified by 1% agarose gel electrophoresis. Sufficient cDNA for subsequent transcriptome sequencing and qRT-PCR was synthesized through reverse transcription with the PrimeScript™ II 1st Strand cDNA Synthesis Kit (TaKaRa, China).

High-throughput RNA-Seq sequencing was performed on the Illumina HiSeqTM2000 platform. Sequence alignment was conducted using the Star software (v2.7.0d), while QoRTs (v1.3.0) was used for data quality control and analysis. Low-quality reads containing adapters were filtered out, and alignment metrics such as error rate, Q_20_, Q_30_, and GC content of the clean data were recorded. HTSeq (v0.11.2) was utilized to obtain quantitative data over a 5-day period for the axillary buds and sett roots of ZZ2 and ZZ9. Differentially expressed genes (DEGs) between sample groups were identified using DESeq2 (v1.26.0), with selection criteria set to q-value < 0.05 and |log2 Fold Change| > 1.5. Venn diagrams were adopted to identify significantly DEGS unique to ZZ2 and ZZ9.

WGCNA (Langfelder and Horvath, 2008) was employed to conduct a combined analysis of transcriptome and metabolome data, which helped identify modules strongly correlated with plant hormones and determine core genes within these modules. Visualization was performed using Cytoscape (v3.9.1). The WGCNA parameters were set as follows: FPKM > 1, R² cutoff ≥ 0.85, pruning height: 0.25, minimum gene count per module: 30, GS (Gene Significance) > 0.80, and MM (Module Membership) > 0.80.

The R package clusterProfiler (Yu et al., 2012) was used for GO and KEGG enrichment analysis, with *Saccharum hybrid cultivar* selected as the reference species database. The parameters were set as follows: pvalueCutoff = 1, pAdjustMethod = “BH”, minGSSize = 10, maxGSSize = 1,000, and qvalueCutoff = 0.5.

### 2.6 qRT-PCR validation

qRT-PCR was conducted using TB Green^®^ Premix Ex Taq™ II dye (TaKaRa, China) to amplify target fragments of the internal reference gene (*GAPDH*) (Que et al., 2009) and nine genes of interest. Specific primers were designed using NCBI Primer-BLAST, and the primer sequences are provided in **Table 1**. The real-time RT-PCR reactions were conducted on a LightCycler^®^ 480 II (Roche, Swiss Confederation) with the following cycling conditions: 95°C for 2 min; with steps 95°C for 10 s and 60°C for 30 s repeated for 40 cycles. After the reaction, amplification and melting curves were analyzed, and the relative expression levels of target genes were calculated using the 2^-△△Ct^ method. Each qPCR reaction included three biological replicates.

**Table 1:** RT-qPCR primer sequence.

Gene ID	Primer F:5’-3’	Primer R:5’-3’
*GAPDH*	ACCGTCTTTGGCATCAGGAAC	GCTTGGGGCAGAGATAACAAC
*ACCH1*	GGGACCTCTTGCAGATAATGTC	CTCTGGCAATGGTCCATAGAA
*ASNS2*	CCAGAGAACACACCCACAAC	ATGCCACACTAGGACCTCCA
*DCAM*	GTGCTTTGGCCCTTCGGAGTTTTC	AGACGCAGCTGACCACCTA
*LOC104229871*	ACGACCTCTCACTCGTCTTC	ACCTCCTCAGTGATCTTGTGC
*MYB1*	GGAAAGGAGTGGTGGTTGG	CGCTTGGAAGTAGCAGGACAC
*P2C44*	GAAGGGATTCGACCGCTTCA	GGCTATAGCAGCCAGCTACG
*PFPA*	TTATGAGTTGCTGCGAGAGAAG	TATCTCAATGTCGCCCATGTAG
*TBA3*	CCGTGGTGATGTTGTTCCTA	GGGTGGCTGATAGTTGATGC
*WRK71*	AAACTTCGGTGGTTGGCGTA	CCTGGTAGAAGAGCTTGCCC

## 3 Results

### 3.1 Asynchronous development in sugarcane stem cuttings: preferential sprouting in ZZ2 and preferential rooting in ZZ9

**Figure 1:**
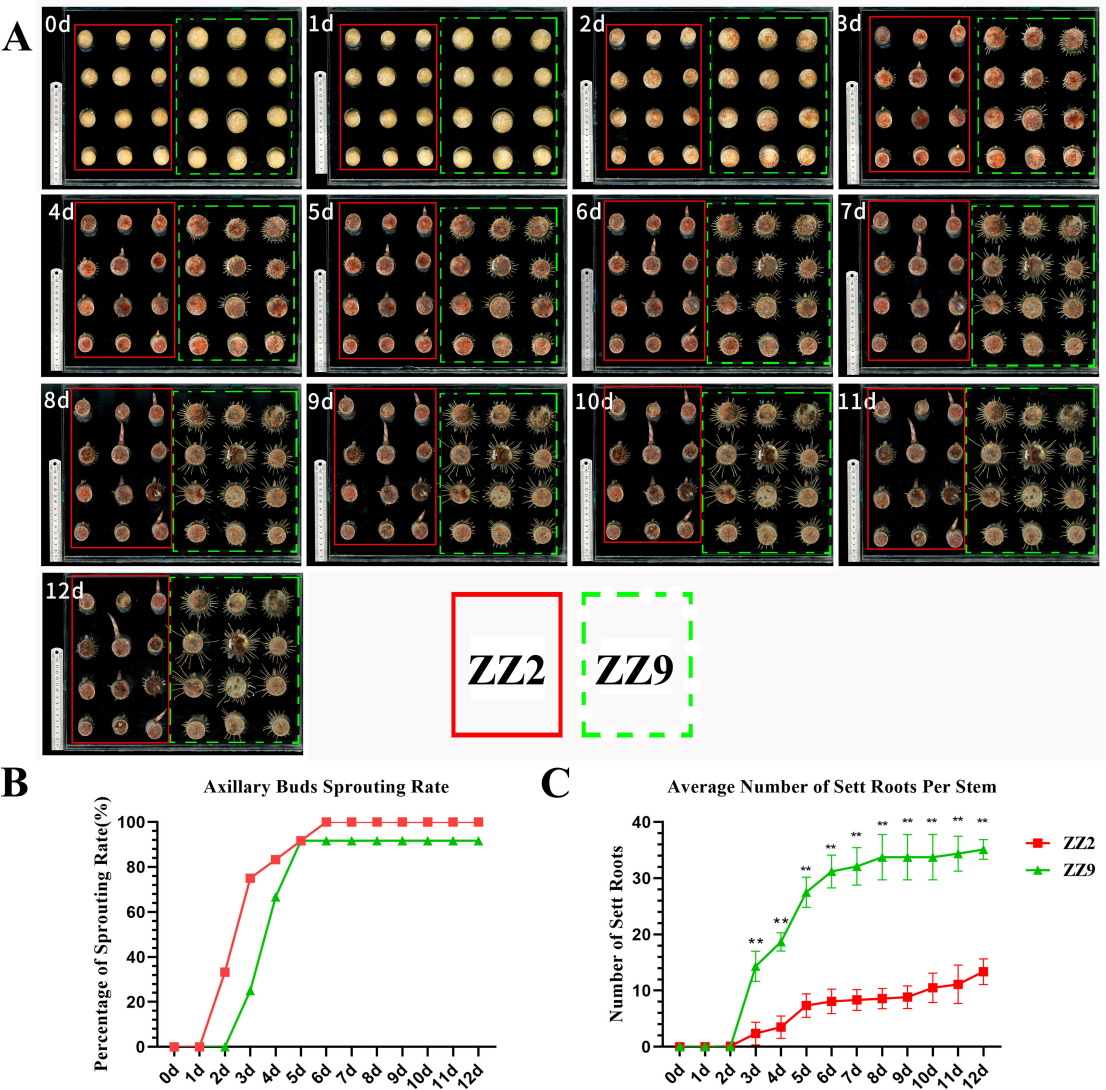
Observation and statistical chart of stem sprouting of ZZ2 and ZZ9 species. **(A)** Time course of shoot emergence. The solid red box and dashed yellow box represent ZZ2 and ZZ9, respectively. **(B)** Sprouting rates of ZZ2B and ZZ9B for 12 days. **(C)** Average number in ZZ2R and ZZ9R for 12 days. **indicates highly significant differences (p ≤ 0.01) between treatments based on the significance test.

On day 2 of cultivation, ZZ2B broke dormancy, with the bud points gradually swelling and the bud scales loosening. In contrast, ZZ9B remained dormant, although the root points began to sprout. By day 3, ZZ2B had broken through the bud scales, forming visible bud tips, and the root points had become active. ZZ9R started elongating, with some bud points gradually swelling and the bud scales loosening. As cultivation progressed, ZZ2B continued to develop and grow, while the sett roots ceased elongating, maintaining a fixed length. On the other hand, the elongation rate of ZZ9B stagnated, but the sett roots continued to develop and elongate. By day 8, the development of axillary buds and sett roots in both ZZ2 and ZZ9 had stabilized with no significant changes (**Figure 1A**).

By analyzing and quantifying the sprouting rate and root formation over a 12-day period in sugarcane stem cuttings, we observed that the most significant changes in sprouting rate for ZZ2 and ZZ9 occurred between days 2 and 5. ZZ2 showed visible bud emergence by day 2, reached a sprouting rate of 33.3%. ZZ9 only began sprouting on day 3, with a sprouting rate of 25.0%, whereas ZZ2 had already reached 75.0%. By day 6, ZZ2 achieved a 100% sprouting rate, while ZZ9 reached 91.7% (**Figure 1B**).

Root primordia in both ZZ2 and ZZ9 began sprouting on the second day. By the third day, the average number of roots in ZZ9 had reached 14.33, whereas in ZZ2, it was significantly lower at 2.375. The root number in ZZ9 was significantly higher than that in ZZ2. Between days 3 and 5, ZZ9 exhibited the most substantial increase in root number. By the eighth day, root sprouting in ZZ9 had stabilized, reaching an average of 35.125 roots by day 12. In contrast, the average root number in ZZ2 continued to increase gradually and stabilized at 13.375 roots on day 12 (**Figure 1C**).

In summary, under identical conditions of temperature and humidity, ZZ2 stem cuttings exhibit earlier bud sprouting, while ZZ9 develops sett roots earlier.

### 3.2 Changes in soluble substances during sprouting of ZZ2 and ZZ9 stem cuttings

**Figure 2:**
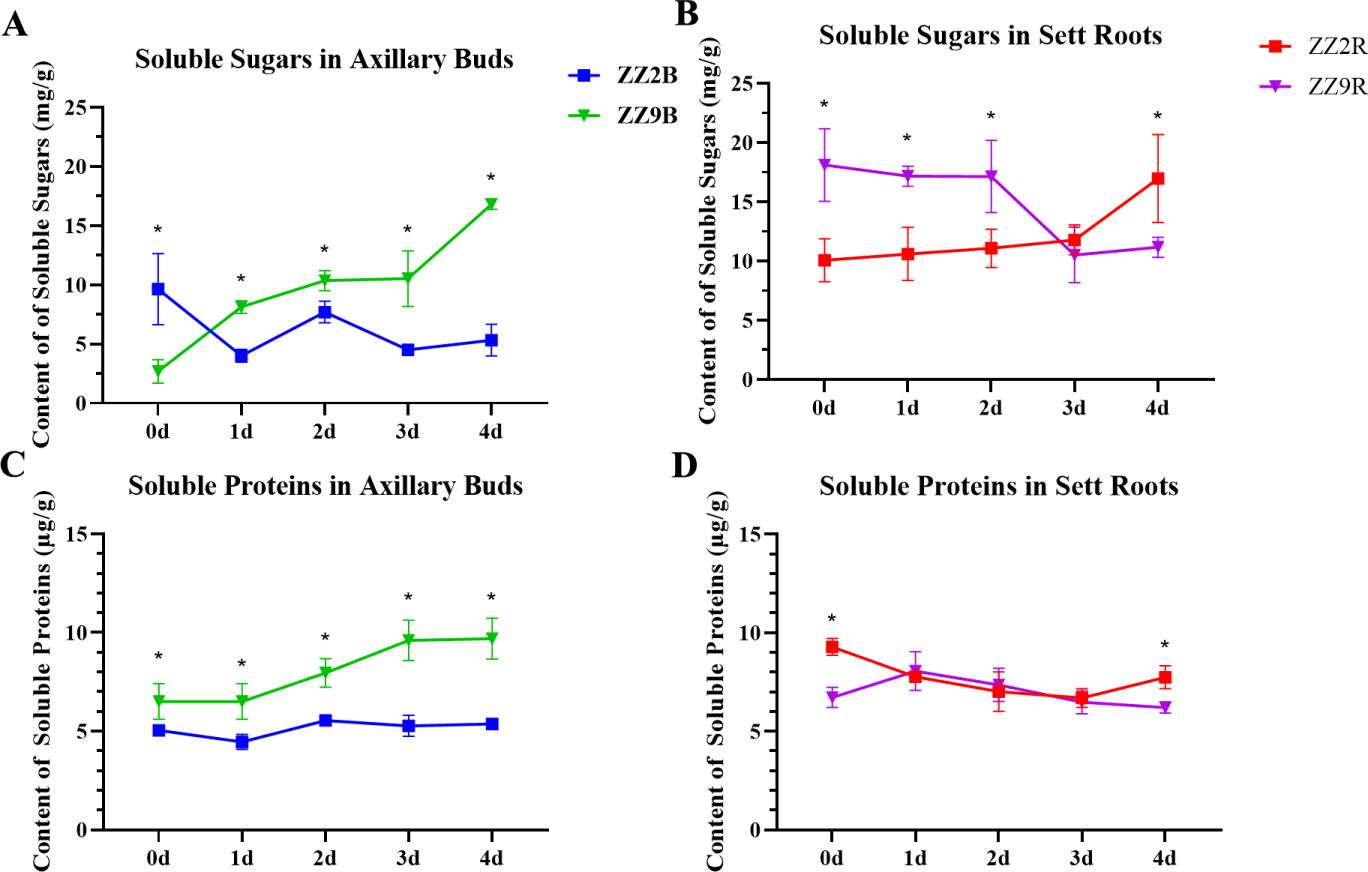
Changes in soluble matter content of ZZ2 and ZZ9 stems. **(A)** Concentration of soluble sugars in axillary buds; **(B)** Concentration of soluble sugars in sett roots; **(C)** Concentration of proteins in axillary buds; **(D)** Concentration of soluble proteins in sett roots. *indicates significance at p ≤ 0.05 based on the significance test.

The soluble sugar content in ZZ2B showed a decreasing trend during development. It started at 9.64 mg/g when single-node cuttings were obtained and dropped to 5.33 mg/g by day 4. Conversely, the soluble sugar content in ZZ9B displayed an increasing trend. It rose from an initial 2.70 mg/g to 16.80 mg/g by day 4. Thus, soluble sugar levels in ZZ2B were significantly higher than those in ZZ9 at the beginning, but the soluble sugar content in ZZ2B became significantly lower than those in ZZ9B as cultivation progressed (**Figure 2A**).

The soluble sugar content in ZZ2R exhibited an increasing trend. It improved from 10.06 mg/g at the start of cultivation to 16.97 mg/g by day 4. On the contrary, the soluble sugar content in ZZ9R showed a decreasing trend. It dropped from 18.12 mg/g on day 0 to 11.17 mg/g by day 4. From days 0 to 2, the soluble sugar content in ZZ2R was significantly lower than those in ZZ9R. However, starting on day 3, ZZ2R gradually surpassed ZZ9R in soluble sugar content, which reached a significant difference by day 4 (**Figure 2B**).

The soluble protein content in ZZ2B remains relatively stable, while ZZ9B showed a more pronounced increase in soluble protein levels. Throughout sprouting, the soluble protein content in ZZ2B remained consistently lower than those in ZZ9B (**Figure 2C**). In ZZ2R, soluble protein content first decreased and then increased. It dropped from 9.28 mg/g on day 0 to 6.47 mg/g by day 3, but rose again to 7.74 mg/g on day 4. Conversely, ZZ9R exhibited an initial increase followed by a decline in soluble protein content. It improved from 6.72 mg/g at day 0 to 8.06 mg/g on day 1, and then steadily decreased to 6.21 mg/g by day 4.

The soluble protein content in ZZ2R and ZZ9R showed significant differences only on days 0 and 4, with no significant differences observed between ZZ2R and ZZ9R from days 1 to 3 (**Figure 2D**).

The spatial distribution of soluble sugars differed significantly between the two sugarcane varieties. Before cultivation, soluble sugars were more concentrated in ZZ2B and ZZ9R. This condition provided a nutritional foundation for the earlier bud sprouting of ZZ2 and the earlier root development of ZZ9. Moreover, the utilization trends of soluble sugars in different tissues align with the observed phenomenon of ZZ2 sprouting earlier while ZZ9 develops roots earlier.

### 3.3 Structural differences in sett roots between ZZ9 and ZZ2

**Figure 3:**
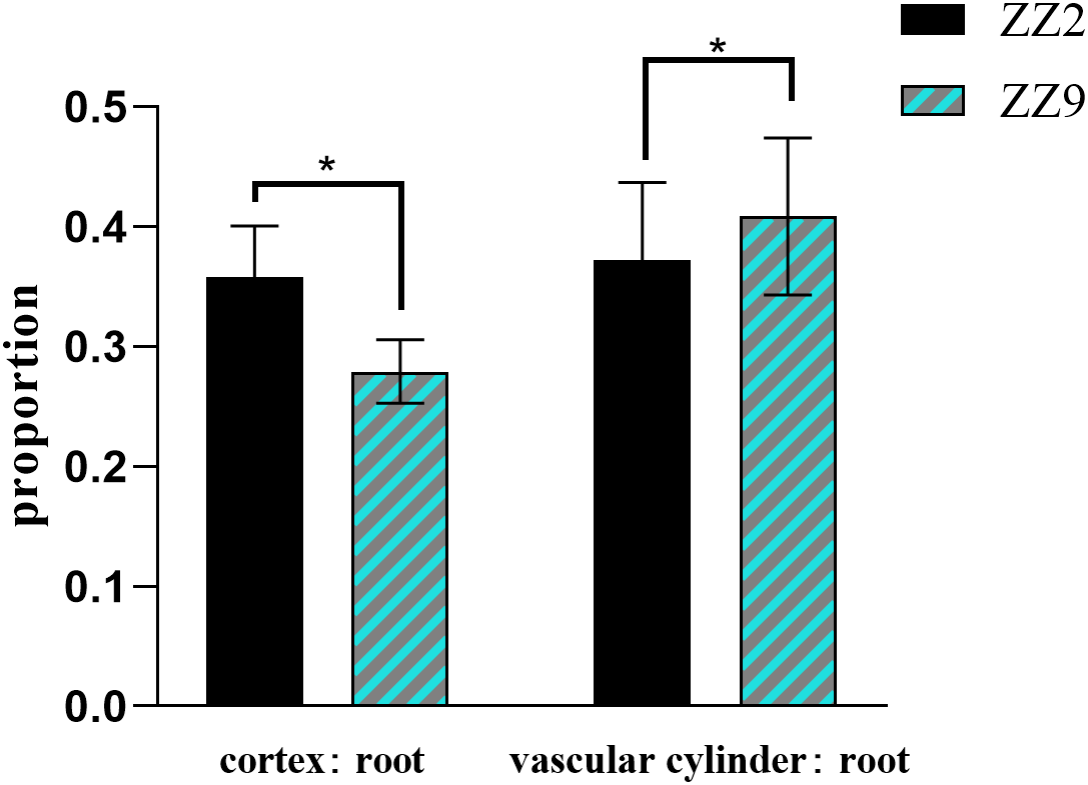
Ratio of root cortex, vascular column, and root diameter of ZZ2 and ZZ9. * indicates significant differences (p ≤ 0.05).

**Figure 4:**
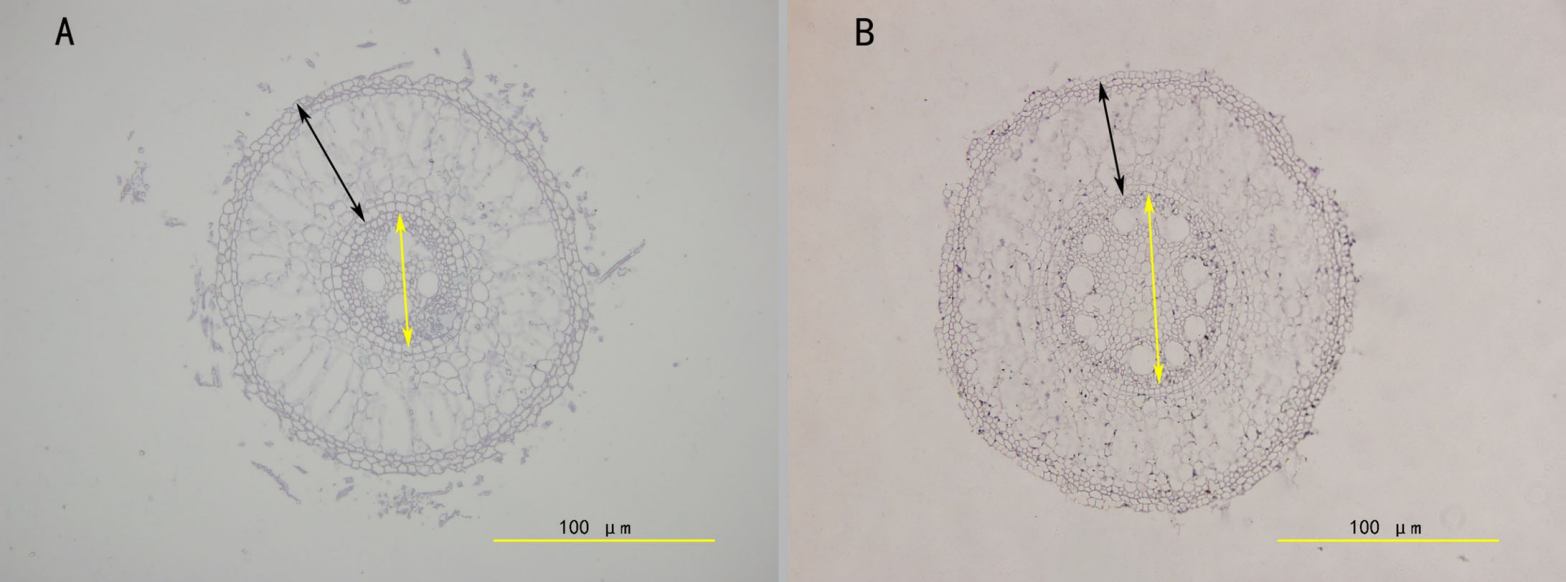
Comparison of the structure of the sett root between ZZ2 and ZZ9. **(A)** ZZ2; **(B)** ZZ9. The cross section of the sett root was done by embedding the root in paraffin and sectioned with a LEICA RM2255 automatic microtome. Images were taken with an Olympus BX63 microscope; black two-way arrows indicate cortical thickness, and yellow two-way arrows indicate vascular column diameter.

Paraffin sections and microscopic observations were conducted on 40 root samples from ZZ2R and ZZ9R. Using ImageJ (Arena et al., 2017) software, the cortical thickness, vascular column diameter, and root diameter of root cross-sections were measured, followed by statistical analysis of significance using GraphPad Prism v8.0 software. The results showed that ZZ2 had a thicker cortex-to-root area ratio, while its vascular-to-root area ratio was thinner (**Figure 3**). In addition, ZZ9R displayed more cavity structures, which indicated an earlier maturation (**Figure 4**).

### 3.4 Metabolomic profiling and multivariate statistical analysis of phytohormones in sugarcane

In this experiment, UHPLC–MS/MS was employed to analyze and detect 24 types of phytohormones across 60 samples from ZZ2 and ZZ9. The lower limits of detections (LLODs) for the target phytohormones ranged between 0.0038 and 7.8125 nmol/L, while the lower limits of quantifications (LLOQs) ranged between 0.0076 and 15.6250 nmol/L (**Supplementary Table S1**). The correlation coefficients (R²) for all target compounds were greater than 0.9972, which suggested a strong quantitative relationship between chromatographic peak areas and compound concentrations. This condition meets the requirements for targeted metabolomics analysis. The QC samples were injected six times. The average recovery rates for all target compounds ranged from 84.2% to 118.9%, with relative standard deviations (RSDs) below 21.1% (**Supplementary Table S2**). These results demonstrate that the method can accurately and reliably detect the target hormone concentrations within the specified range.

Based on the preprocessed hormone detection data, 19 phytohormones were selected for further statistical analysis. PLS-DA was performed on each pair of samples, and phytohormones with VIP values ≥ 1 (**Supplementary Table S3**) were selected. The phytohormones with consistent differences after three or more days of cultivation are listed in **Table 2**.

**Table 2:** Differential metabolites in root and shoot tissues.

Tissue	Plant Hormone
axillary bud	3-Indolebutyric acid
	N6-isopentenyladenosine
	Gibberellin A7
	cis-Zeatin
sett root	Abscisic acid
	Gibberellin A3
	N6-isopentenyladenosine
	Gibberellin A7
	N-((-)-jasmonoyl)-S-isoleucine
	(±)-Jasmonic acid

**Figure 5:**
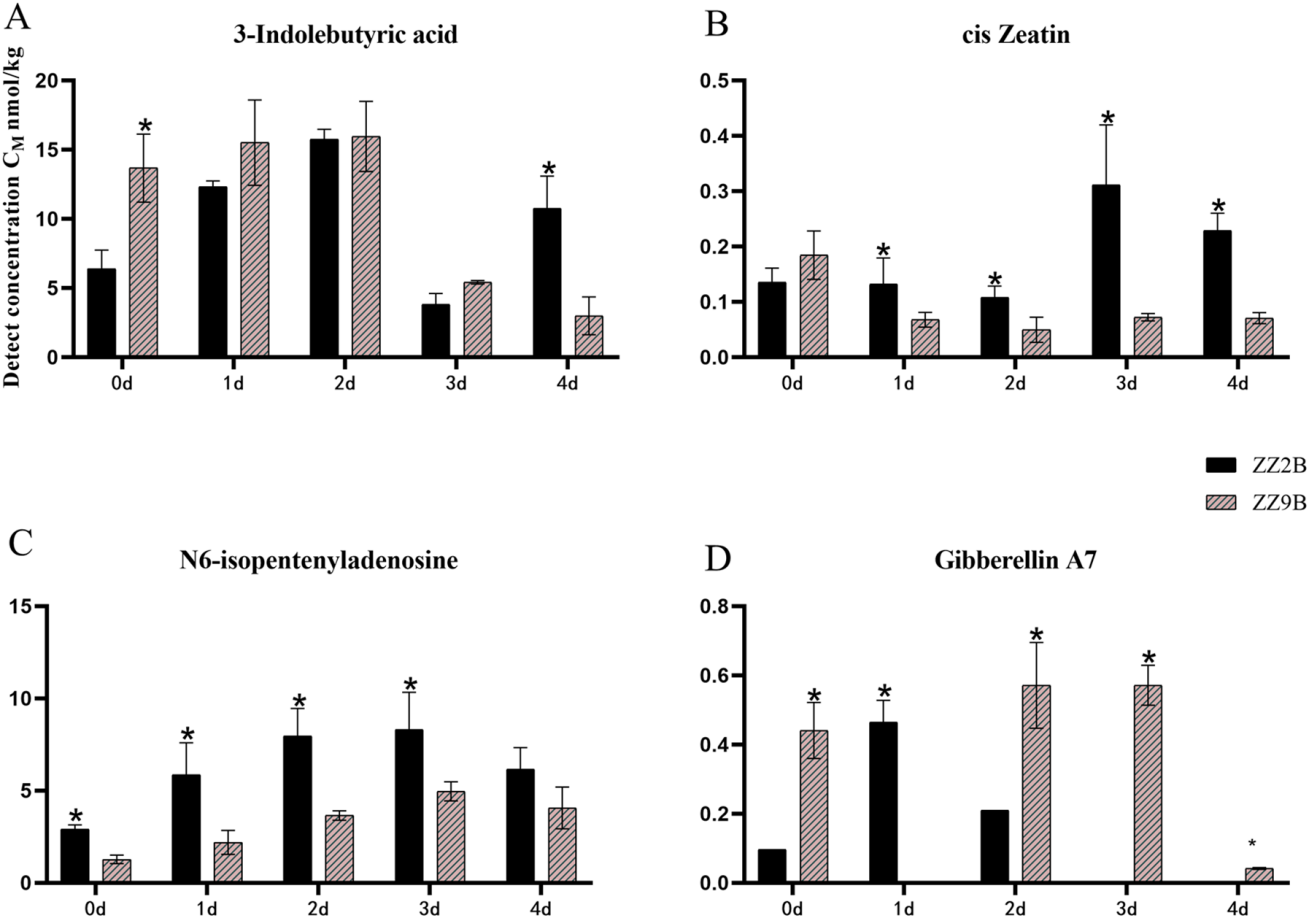
Comparison of hormone concentration in axillary buds between ZZ2 and ZZ9. **(A)** The detection concentration of IBA in ZZ2B and ZZ9B. **(B)** The detection concentration of cZ in ZZ2B and ZZ9B. **(C)** The detection concentration of IPA in ZZ2B and ZZ9B. **(D)** The detection concentration of GA7 in ZZ2B and ZZ9B. The x-axis represents the time points of hormone detection, and the y-axis represents the detected concentration of the target hormone. Both are represented in units of nmol/kg. The asterisk (*) indicates significant differences where VIP ≥ 1 for the same day.

**Figure 6:**
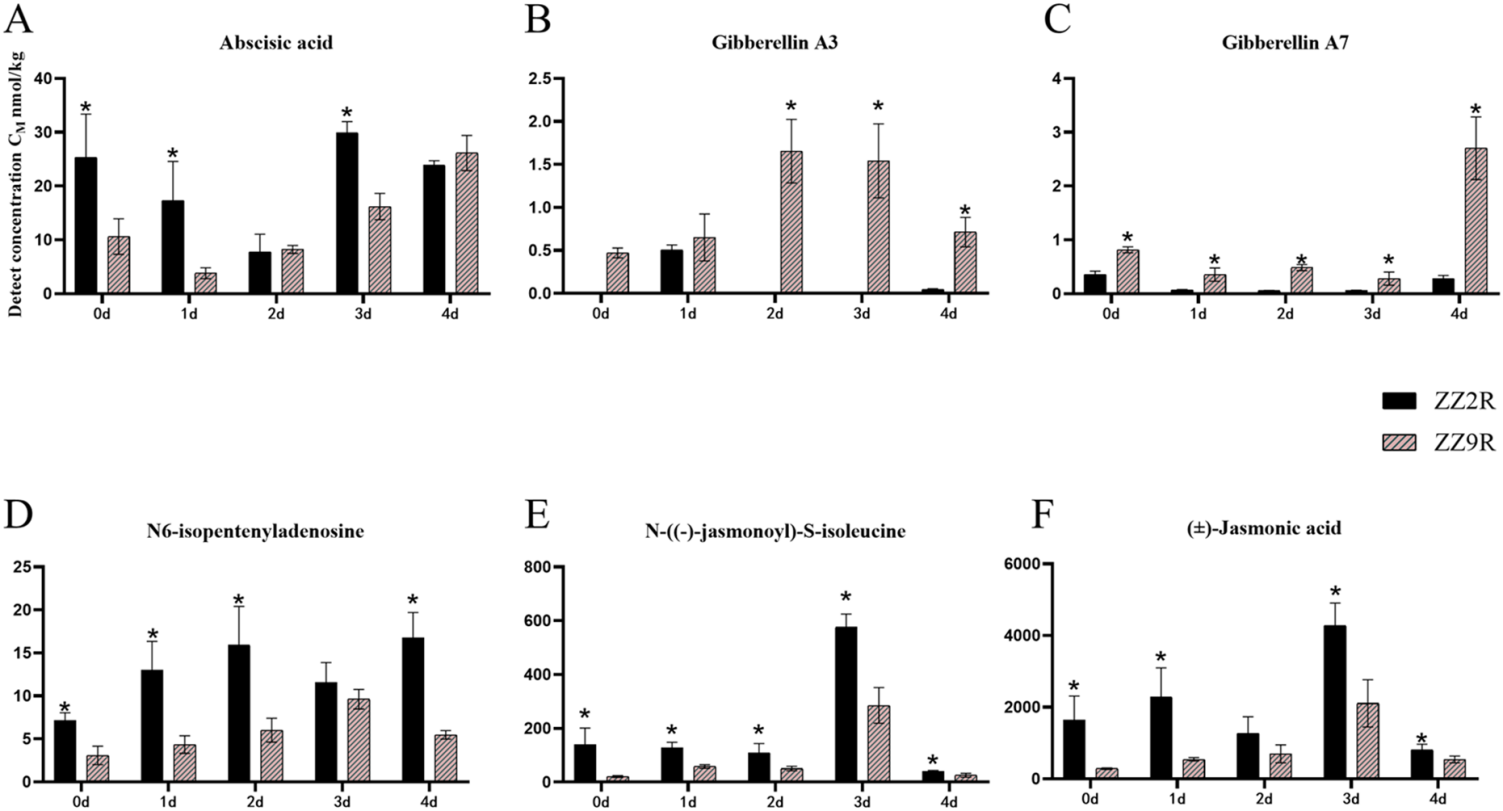
Comparison of hormone concentration in sett roots between ZZ2 and ZZ9. **(A)** The detection concentration of ABA in ZZ2R and ZZ9R. **(B)** The detection concentration of GA3 in ZZ2R and ZZ9R. **(C)** The detection concentration of GA7 in ZZ2B and ZZ9B. **(D)** The detection concentration of IPA in ZZ2R and ZZ9R. **(E)** The detection concentration of JA-Ile in ZZ2R and ZZ9R. **(F)** The detection concentration of JA in ZZ2R and ZZ9R. The x-axis represents the time points of hormone detection, and the y-axis represents the detected concentration of the target hormone. Both are represented in units of nmol/kg. The asterisk (*) indicates significant differences where VIP ≥ 1 for the same day.

The differential hormones in ZZ2B and ZZ9B were identified as Auxins (IBA), Cytokinins (cZ, IPA), and Gibberellins (GA_7_) (**Figure 5**). In the sett roots, the differential hormones included ABA, Gibberellins (GA_3_, GA_7_), Cytokinins (IPA), and Jasmonates (JA-Ile, JA) (**Figure 6**).

To verify the reliability of the PLS-DA analysis model, a PLS-DA permutation test was performed on each group, with multiple (N=999) corresponding PLS-DA models generated to obtain the R²Y and Q² values for random models. The results indicated that none of the analysis models for any group exhibited overfitting, which supported the reliability of the differential hormone selection based on VIP ≥ 1 (**Supplementary Figure S1**).

The detection results for differential metabolite hormone concentrations at five time points in ZZ2B and ZZ9B indicated that IBA was differentially expressed in axillary bud tissues. During the early cultivation period, IBA concentration was lower in ZZ2B but surpassed that of ZZ9 by day 4 of cultivation (**Figure 5A**). In addition, cZ and IPA exhibited cytokinin activity and had functional similarities or complementary roles, which displayed similar expression patterns across different experimental groups. The concentrations of cZ and IPA were generally higher in ZZ2B. And showed an upward trend. On the third day, the cZ content in ZZ2B was significantly higher than those in ZZ9B (**Figures 5B, C**). The daily GA_7_ content in the axillary buds of the two varieties showed distinct opposite trends. Specifically, The daily changes in GA_7_ content in the axillary buds of the two varieties exhibit completely opposite trends. In ZZ2B, the changes are more pronounced on days 1 and 2, whereas in ZZ9B, they are more evident on days 2 and 3 (**Figure 5D**).

The detected concentrations of differential metabolite hormones in ZZ2R and ZZ9R over five time points showed that ABA levels in ZZ9R were significantly lower than those in ZZ2R. ABA reached its lowest level on the first day of cultivation ZZ9R, while the lowest level occurred on the second day ZZ2R. Overall, the ABA content in both varieties followed a trend of initial decline followed by an increase (**Figure 6A**). The GA_3_ content in ZZ9R is significantly higher than that in ZZ2R on days 2, 3, and 4. Additionally, the GA_7_ content in ZZ9R is significantly higher than in ZZ2R across all five time points (**Figure 6B, C**). Meanwhile, IPA levels were significantly lower in ZZ9 than in ZZ2 (**Figure 6D**). JA-Ile and JA are both active forms of jasmonic acid and exhibit similar expression patterns across different experimental groups. The JA levels in ZZ9R were consistently lower than those in ZZ2R, with both reaching their peak levels on the third day of cultivation (**Figure 6E, F**).

### 3.5 Differentially expressed genes in axillary buds and sett roots of ZZ2 and ZZ9 during sprouting

Given that ZZ2 and ZZ9 show the most significant differences in root and bud sprouting rates within the first four days of cultivation, the two varieties may exhibit heterochrony at the level of gene transcription. Analyzing the DEGs can directly reveal the distinctions between them.

The average Q_30_ value for base detection in the transcriptomes of in the buds and roots of ZZ2 and ZZ9 was above 94% (**Supplementary Table S4**), which suggested good sequencing quality and reliable data. In general, a total alignment rate above 70% against the reference genome suggests no contamination. In this study, the average alignment rate for the 60 sample materials was over 90% (**Supplementary Table S5**), which implied no contamination issues and confirmed the suitability of the selected reference genome.

**Figure 7:**
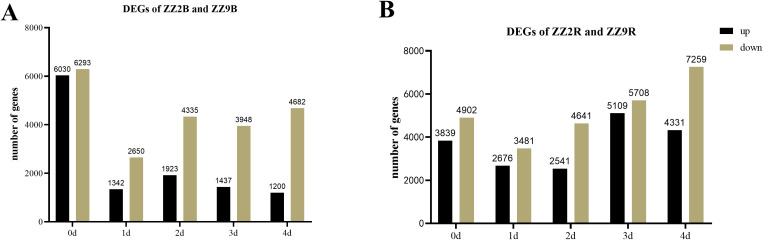
Statistics of DEGs in the bud and root tissues of ZZ2 and ZZ9. **(A)** DEGs in axillary buds; **(B)** DEGs in sett roots.

The differential gene expression in ZZ2 compared with ZZ9 in axillary buds (with significance at padj < 0.01) is shown in **Figure 7A**: Initially, 12,323 genes showed significant differential expression, with 6,030 genes upregulated and 6,293 genes downregulated in ZZ2B. On day 1, 3,992 genes were differentially expressed, with 1,342 upregulated and 2,650 downregulated genes. On day 2, 6,258 genes were differentially expressed, including 1,923 upregulated and 4,335 downregulated genes. On day 3, 5,385 genes showed differential expression, with 1,740 upregulated and 3,645 downregulated genes. By day 4, 5,882 genes were differentially expressed, with 1,200 upregulated and 4,682 downregulated genes.

In ZZ9 compared with ZZ2, the initial analysis revealed 8,741 genes with significant differential expression in sett roots, with 4,902 upregulated genes and 3,839 downregulated genes. On day 1, 6,157 genes were differentially expressed, including 3,481 upregulated and 2,676 downregulated genes. On day 2, 7,182 genes showed differential expression, with 4,641 upregulated and 2,541 downregulated. On day 3, 10,817 DEGs were detected, with 5,708 upregulated and 5,109 downregulated genes. By day 4, 11,590 genes were differentially expressed, with 7,259 upregulated and 4,331 downregulated genes (**Figure 7B**).

**Figure 8:**
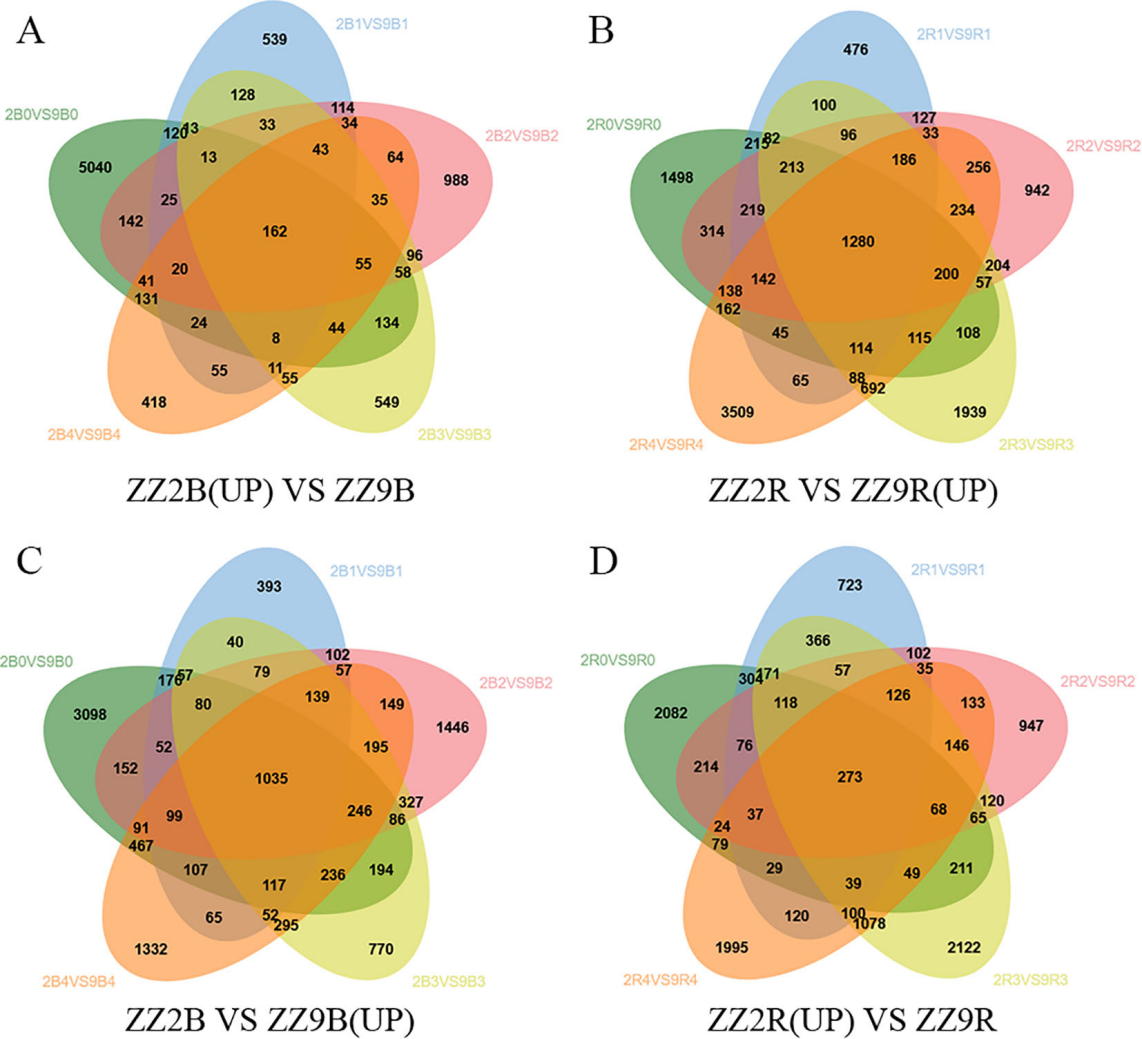
Venn diagram of DEGs in ZZ2B and ZZ9R. **(A)** A Set of Genes with Significantly Upregulated Expression Specific to ZZ2B. **(B)** A Set of Genes with Significantly Upregulated Expression Specific to ZZ9R. **(C)** A Set of Genes with Significantly Downregulated Expression Specific to ZZ2B. **(D)** A Set of Genes with Significantly Downregulated Expression Specific to ZZ9R.

In ZZ2B, 162 genes were uniquely and significantly upregulated (**Figure 8A**), while 1,035 genes were uniquely downregulated (**Figure 8C**). In ZZ9R, 1,280 genes were uniquely and significantly upregulated (**Figure 8B**), and 273 genes were uniquely downregulated (**Figure 8D**).

GO and KEGG enrichment analyses were performed on the genes that were significantly upregulated and downregulated in ZZ2B and ZZ9R.

**Figure 9:**
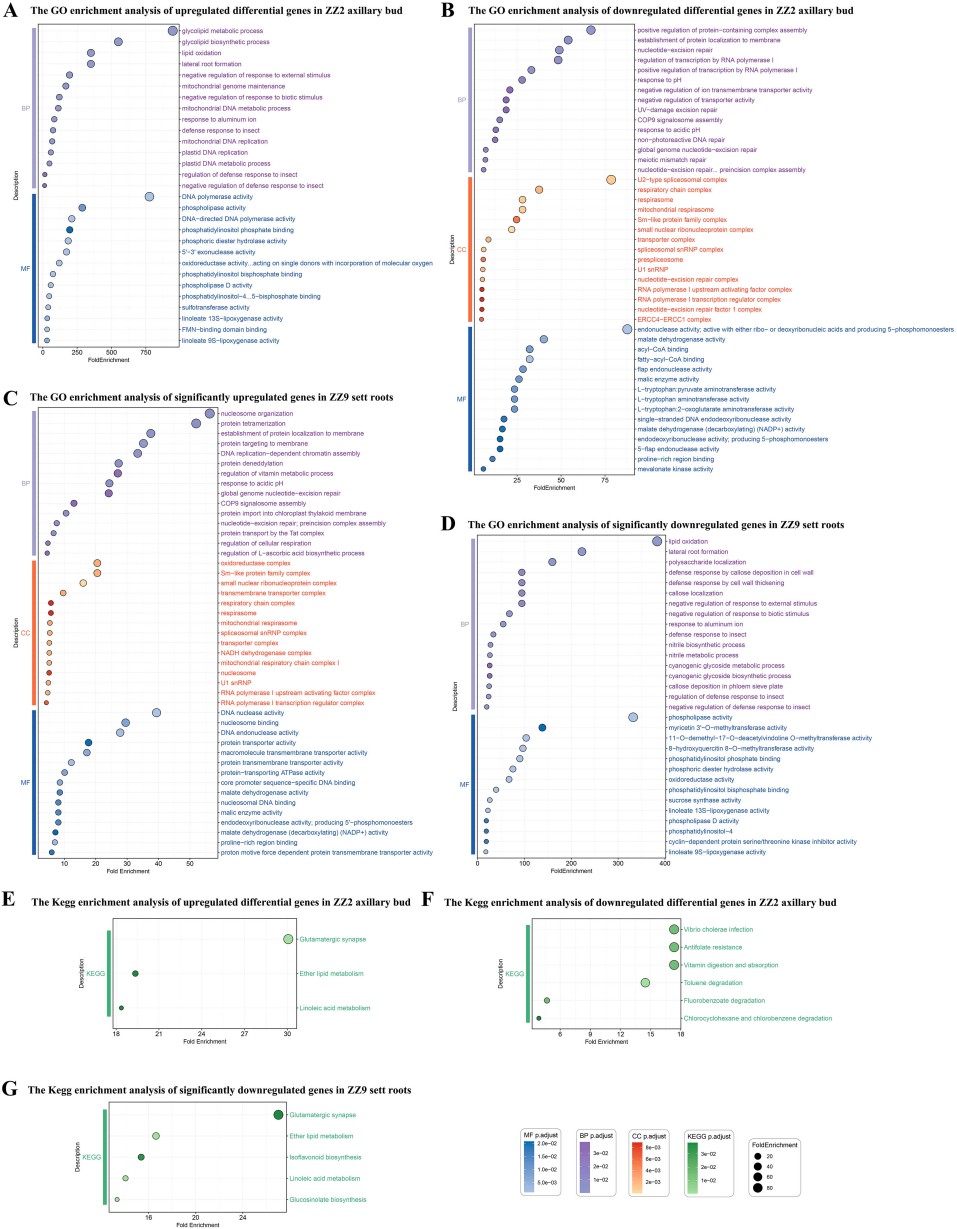
GO and KEGG enrichment results of DEGs in the bud and root tissues of two sugarcane varieties. **(A)** GO enrichment analysis of upregulated differentially expressed genes in ZZ2B. **(B)** GO enrichment analysis of downregulated differentially expressed genes in ZZ2B. **(C)** GO enrichment analysis of significantly upregulated genes in ZZ9R. **(D)** The GO enrichment analysis of significantly downregulated genes in ZZ9R. **(E)** KEGG enrichment analysis of upregulated differentially expressed genes in ZZ2B. **(F)** KEGG enrichment analysis of downregulated differentially expressed genes in ZZ2B. **(G)** KEGG enrichment analysis of significantly downregulated genes in ZZ9R.

GO enrichment analysis of upregulated differentially expressed genes in ZZ2B during sprouting primarily focused on the following key aspects (**Figure 9A**): defense response regulation (GO:1900366, GO:2000068, GO:0002213), metabolic processes and metabolic regulation (GO:0034440, GO:0016702, GO:0046857, GO:0004630, GO:0004620, GO:0033258, GO:0033259, GO:0006264, GO:0003887, GO:0008409), root development and environmental stress response (GO:0010311, GO:0016036), transferase and reductase activity (GO:0008146, GO:0016782).

KEGG enrichment analysis of upregulated DEGs during the sprouting of ZZ2B was enriched in ko00591, ko00565, and ko04724 (**Figure 9E**).

GO enrichment analysis of downregulated differentially expressed genes during the sprouting of ZZ2B mainly involved the following key aspects (**Figure 9B**): DNA repair and nucleic acid metabolism (GO:0006294, GO:0070911, GO:0000710), protein assembly and membrane localization (GO:0031334, GO:0072657), transcription regulation and RNA processing (GO:0045943, GO:0005685, GO:0071010), membrane transport and ion metabolism (GO:0034766, GO:0090575), mitochondrial respiration and ATP synthesis (GO:0005746, GO:0016469), cytoskeleton and structural proteins (GO:0030017, GO:0048256), and metabolic enzymes and cofactor binding (GO:0004470, GO:0000253, GO:0000062).

KEGG enrichment analysis of downregulated DEGs during the sprouting of ZZ2B was enriched to ko04977, ko00361, ko00364, ko00623, ko01523, and ko05110 (**Figure 9F**).

GO enrichment analysis of significantly upregulated genes in ZZ9R mainly involved the following key biological processes and cellular functions (**Figure 9C**): DNA repair and chromatin-related processes (GO:0006294, GO:0070911, GO:0006335, GO:0034728), protein transport (GO:0043953, GO:0045038, GO:0006612, GO:0072657, GO:0044743, GO:0065002), transcription regulation and RNA metabolism (GO:0045943, GO:0006356, GO:0030532, GO:0005685, GO:0071010), energy metabolism and respiratory chain (GO:0045271, GO:0005747, GO:0005746, GO:0009631), cell cycle regulation (GO:0010971, GO:1902751) and stress and environmental response (GO:0010447, GO:0009268, GO:0010044), and enzyme activity and metabolic regulation (GO:0016615, GO:0004470, GO:2000082).

GO enrichment analysis of significantly downregulated genes in ZZ9R mainly involved the following key biological processes (**Figure 9D**): plant defense responses and regulation (GO:0002213, GO:0052542, GO:1900366, GO:0032102), metabolic processes of secondary metabolites (GO:0009821, GO:0019756, GO:0009074, GO:0006569), cell wall-related responses (GO:0052542, GO:0052386), redox and lipid metabolism (GO:0034440, GO:0016165, GO:0016701), and hormone and signal transduction (GO:0009851).

KEGG enrichment analysis of downregulated DEGs during the sprouting of ZZ9R was enriched to ko00591, ko00565, ko04724, ko00943, and ko00966 (**Figure 9G**)

### 3.6 WGCNA of transcriptome and metabolome data during sprouting in two sugarcane varieties

The complete gene sets in the axillary buds and sett roots of ZZ2 and ZZ9 were subjected to data preprocessing. Weighted gene co-expression networks were then constructed separately for each. The soft threshold of the network and the results from the scale-free topology test met the scale-free criterion (**Supplementary Table S6**).

A co-expression network constructed from the genes in ZZ2B identified 34 color modules (**Supplementary Table S7**). A hierarchical clustering dendrogram of ZZ2B illustrates the results of each module (**Supplementary Figure S2A**). Analysis of the correlation between gene modules in ZZ2B and traits revealed a high positive correlation of the salmon module with GA_7_ (r = 0.88, p = 0.00006), and a strong positive correlation of the yellow module with GA_3_ (r = 0.92, p = 0.0000088). In addition, the yellow module was highly positively correlated with DL-Dihydrozeatin (r = 0.86, p = 0.00015) (**Supplementary Figure S2B**). GO and KEGG enrichment analyses were performed on the gene sets of the salmon and yellow modules to investigate the roles of GA_7_, GA_3_, and DL-Dihydrozeatin in promoting preferential sprouting in ZZ2R.

**Figure 10:**
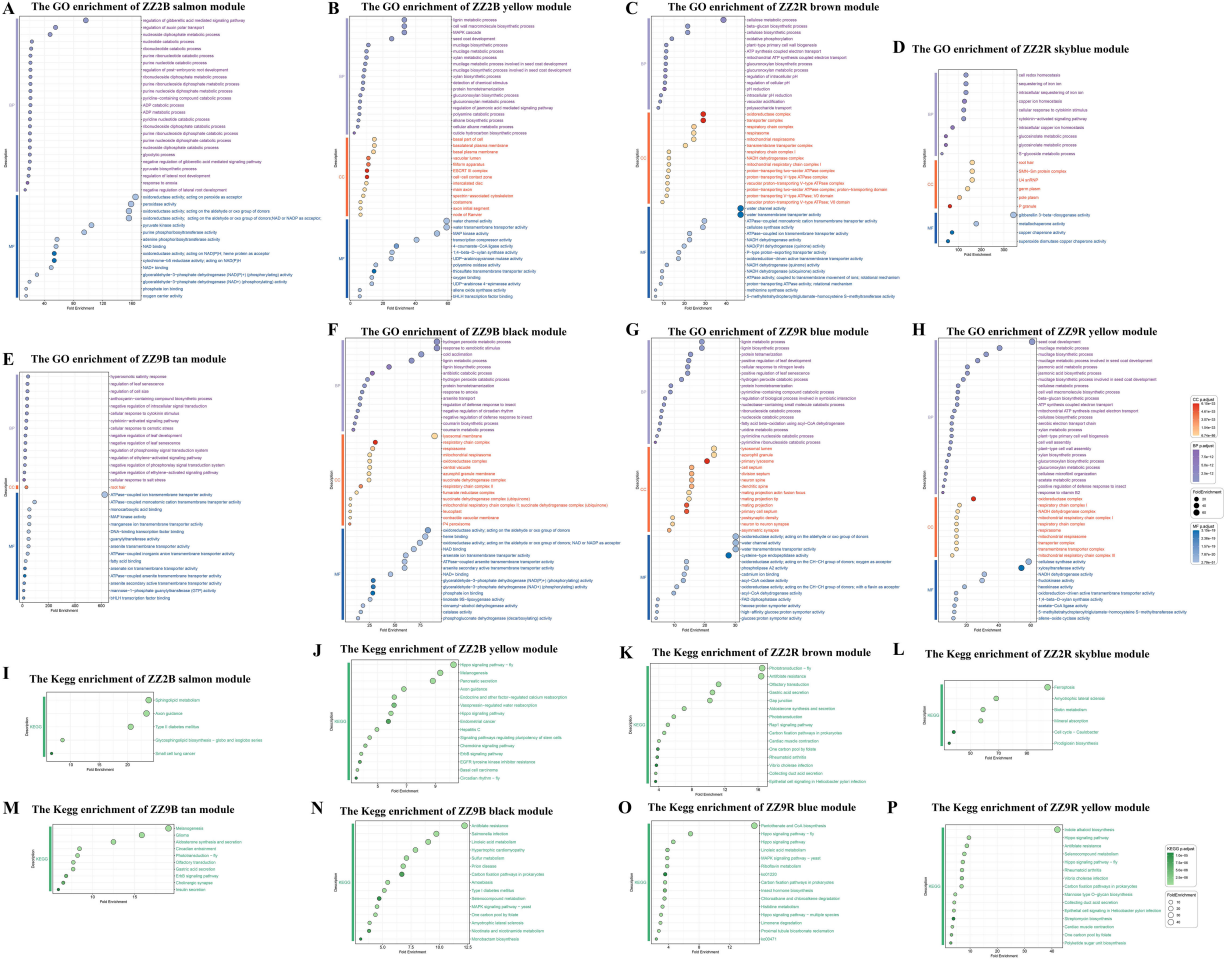
GO and KEGG enrichment results of WGCNA module in the axillary buds and sett roots of ZZ2 and ZZ9. **(A)** GO enrichment in the salmon module of ZZ2B. **(B)** GO enrichment of the yellow module of ZZ2B. **(C)** GO enrichment of the brown module of ZZ2R. **(D)** GO enrichment in the skyblue module of ZZ2R. **(E)** GO enrichment in the tan module of ZZ9B. **(F)** GO enrichment of the black module of ZZ9B. **(G)** GO enrichment in the blue module ZZ9R. **(H)** GO enrichment in the yellow module of ZZ9R. **(I)** KEGG enrichment in the salmon module of ZZ2B. **(J)** KEGG enrichment in the yellow module of ZZ2B. **(K)** KEGG enrichment in the brown module of ZZ2R. **(L)** KEGG enrichment in the skyblue module of ZZ2R. **(M)** KEGG enrichment in the tan module of ZZ9B. **(N)** KEGG enrichment in the black module of ZZ9B. **(O)** KEGG enrichment in the blue module of ZZ9R. **(P)** KEGG enrichment in the yellow module of ZZ9R.

GO enrichment analysis results for the genes in the salmon module in ZZ2B mainly involved the following key biological processes: energy metabolism processes (GO:0006096, GO:0009134, GO:0009137, GO:0009181, GO:0009191, GO:0019364, GO:0046031, GO:0046032, GO:0009135, GO:0009179, GO:0009185, GO:0006195, GO:0009154, GO:0009261, GO:0009166, GO:0009132), cytoskeletal regulation (GO:0030042, GO:0030041, GO:0051017, GO:1901880, GO:0031333), embryo and root development (GO:0001824, GO:2000023, GO:0010311, GO:1901332), oxygen and gas transport (GO:0015671, GO:0015669, GO:0005344); responses to environmental factors and stress (GO:0034059, GO:0010286), and cellular signal transduction (GO:0009937, GO:2000012) (**Figure 10A**).

KEGG gene of the salmon module in ZZ2B was enriched to ko04930, ko05222, ko00603, ko04360, and ko00600 (**Figure 10I**)

GO enrichment analysis results for the genes in the yellow module of ZZ2B mainly involved the following key biological processes: metabolic processes (GO:0006023, GO:0006024, GO:0006063, GO:0009310, GO:0009695, GO:0006006, GO:0009825, GO:0010876, GO:0034434, GO:0034389, GO:0031539, GO:0006576, GO:0009695, GO:0009694), stress responses and signal transduction (GO:0002220, GO:0009609, GO:0009757, GO:0032490, GO:0070483, GO:0071467, GO:0072595), hormone signaling (GO:0009727, GO:0009739, GO:0009734); defense and immune responses (GO:0002446, GO:0006955, GO:0012501), cellular and molecular transport (GO:0006814, GO:0035725, GO:0015709, GO:0048193, GO:0048280); cell cycle and division (GO:0051301, GO:0000278, GO:0007010), development and morphogenesis (GO:0048366, GO:0010015, GO:0048468, GO:0030036), protein modification and degradation (GO:0006468, GO:0016567, GO:0019941, GO:0006511), regulation of gene expression and transcription (GO:0006351, GO:0006260, GO:0006281), and cell death and aging (GO:0012501, GO:0008219) (**Figure 10B**).

KEGG gene of the yellow module in ZZ2B was enriched to ko01521, ko04711, ko04390, ko05160, ko04962, ko04062, ko04012, ko05217, ko04360, ko04916, ko04961, ko04550, ko04972, ko05213, and ko04391 (**Figure 10J**).

A co-expression network constructed from the genes in ZZ2R identified 32 color-associated modules (**Supplementary Table S7**). A hierarchical clustering dendrogram illustrates the results of each module (**Supplementary Figure S2C**). In the correlation analysis between gene modules in ZZ2R and traits, the brown and skyblue modules showed high positive correlations with trans-Zeatin-riboside, with rates of 0.80 (p = 0.00037) and 0.77 (p = 0.00073), respectively (**Supplementary Figure S2D**). GO and KEGG enrichment analyses were performed on the gene sets of the brown and skyblue modules.

GO enrichment analysis results for the genes in the brown and skyblue modules of ZZ2R mainly involved the following key biological processes: cellular metabolic processes (GO:0006085, GO:0006119, GO:0009060, GO:0042775), substance transport and translocation (GO:0015843, GO:0006833, GO:0010324), stress responses and adaptation (GO:0009407, GO:0042773, GO:0071462, GO:0045454, GO:0097577), cellular structure and motility (GO:0006911, GO:0010325, GO:0051274), signal transduction and regulation (GO:0043181, GO:0051452), metabolic pathways and biosynthesis (GO:0019326, GO:0009853, GO:0046270, GO:0019757, GO:0019760, GO:0016143), gene regulation and developmental processes (GO:0002320, GO:0060993, GO:0048354), molecular transport and ion regulation (GO:0006878, GO:0055070, GO:0006880), signal transduction and regulation (GO:0009736, GO:0071368), and gene regulation and chromatin modification (GO:0005687, GO:0034719, GO:0043186) (**Figures 10C, D**).

KEGG gene of the brown and skyblue modules in ZZ2R seedlings was enriched to ko05120, ko04966, ko05110, ko05323, ko00670, ko04015, ko00720, ko04260, ko04925, ko04744, ko01523, ko04971, ko04540, ko04745, ko04740, ko00333, ko04112, ko04978, ko00780, ko05014, and ko04216 (**Figures 10K, L**).

A co-expression network constructed from the genes in ZZ9B identified 44 color-associated modules (**Supplementary Table S7**). A hierarchical clustering dendrogram illustrates the results of each module (**Supplementary Figure S2E**). In the correlation analysis between gene modules in ZZ9B and traits, the tan module showed a strong positive correlation with Abscisic acid at 0.8 (p = 0.00035), while the black module exhibited a strong negative correlation with 3-Indolebutyric acid at -0.81 (p = 0.00026) (**Supplementary Figure S2F**). GO and KEGG enrichment analyses were performed on the gene sets of the tan and black modules.

GO enrichment analysis results for the genes in the tan module of ZZ9B mainly involved the following key biological processes: response to environmental stress (GO:0071472, GO:0009407), signal transduction and response (GO:0009736, GO:0009862), metabolic processes (GO:0019326, GO:0046256), cellular transport and degradation (GO:0015700, GO:0035618), enzyme activity (GO:0008490, GO:0004364), binding activity (GO:0005544, GO:0005504), membrane and membrane proteins (GO:0015446, GO:0005245), mitochondrial processes (GO:0004475), signaling pathways (GO:0000165, GO:0071368), and secondary metabolism (GO:0042538, GO:0009718) (**Figure 10E**).

KEGG gene of the tan module in ZZ9B was enriched to ko04911, ko04725, ko04971, ko04740, ko04012, ko04745, ko04713, ko04925, ko05214, and ko04916 (**Figure 10M**).

GO enrichment analysis results for the genes in the black module of ZZ9B mainly involved the following key biological processes: carbohydrate metabolism (GO:0006098, GO:0006099, GO:0006101), amino acid metabolism (GO:0006569, GO:0006570, GO:0006521), nucleotide metabolism (GO:0009164, GO:0006099), energy metabolism and electron transport (GO:0006121, GO:0042775, GO:0019682), response to environmental stimuli and immune response (GO:0009410, GO:0002213, GO:0002446, GO:0051703), hormone signaling and signal transduction (GO:0007187, GO:0009608, GO:0007231), mitochondrial and organelle functions (GO:0042773, GO:0045337), cell cycle and growth (GO:0007009, GO:0010200, GO:0051131), transport and transmembrane proteins (GO:0015203, GO:0030775), and enzyme activity-related processes (GO:0016854, GO:0016701, GO:0004616) (**Figure 10F**).

KEGG gene of the black module in ZZ9B was enriched to ko05014, ko04011, ko00760, ko00261, ko00450, ko00670, ko05410, ko05146, ko00920, ko00720, ko00591, ko05132, ko04940, ko05020, and ko01523 (**Figure 10N**).

A co-expression network constructed from the genes in ZZ9R identified 33 color-associated modules (**Supplementary Table S7**). A hierarchical clustering dendrogram illustrates the results of each module (**Supplementary Figure S2G**). In the correlation analysis between gene modules in ZZ9R and traits, the blue module showed a strong positive correlation with ABA, reached 0.84 (p = 0.000091) (**Supplementary Figure S2H**). The yellow module exhibited high positive correlations with CKs (cytokinins), specifically trans-Zeatin-riboside at 0.92 (p = 0.0000016), trans-Zeatin at 0.88 (p = 0.000013), and N6-isopentenyladenosine at 0.85 (p = 0.000054). GO and KEGG enrichment analyses were performed on the gene sets in the blue and yellow modules.

GO enrichment analysis results for the genes in the blue module of ZZ9R mainly involved the following key biological processes: enrichment in metabolic and biosynthetic pathways (GO:0009164, GO:0006216, GO:0009119, GO:0009116), amino acid and lipid metabolism (GO:0006570, GO:0006598, GO:0006635), immune and defense responses (GO:0002444, GO:0002443, GO:0002573, GO:0002263, GO:0002283), cellular stress responses (GO:0006984, GO:0009635, GO:0006970), signal transduction (GO:0007169, GO:0007263, GO:0030850, GO:0007186), cytoskeleton organization and cytokinesis (GO:0000936, GO:0000935, GO:0030036), DNA repair and replication (GO:0006974, GO:0006281, GO:0006260); substance transport and membrane trafficking (GO:0015791, GO:0015749, GO:0015801, GO:0006833) (**Figure 10G**).

KEGG gene in the blue module in ZZ9R seedlings was enriched to ko04011, ko00471, ko00720, ko00591, ko00340, ko04390, ko00625, ko00903, ko04964, ko04391, ko00740, ko04392, ko00770, ko01220, and ko00981 (**Figure 10O**).

GO enrichment analysis results for the genes in the yellow module of ZZ9R primarily involved the following key biological processes: metabolic processes (GO:0005978, GO:0045493, GO:0046365, GO:0006642, GO:0009163, GO:0006166, GO:0006208, GO:0006212, GO:0006083, GO:0006096, GO:0006097, GO:0006730, GO:0006099, GO:0006637, GO:0009062, GO:0006637, GO:0046470), cell cycle and division (GO:0006260, GO:0006261, GO:0007059, GO:0051225, GO:0000922), signal transduction and response (GO:0007165, GO:0007166, GO:0007186, GO:0009414, GO:0009611, GO:0009862, GO:0031668, GO:0071394), cell structure and organization (GO:0005737, GO:0005886, GO:0005764, GO:0000221, GO:0030036), development and differentiation (GO:0048732, GO:0048562, GO:0048468, GO:0007389, GO:0009950, GO:0032501), transport and translocation (GO:0015031, GO:0015767, GO:0015453), stress response and immunity (GO:0006950, GO:0009611, GO:0006955), enzyme activity (GO:0016853, GO:0003843), organelle-related functions (GO:0005739, GO:0005794, GO:0005764), and hormone signaling and regulatory mechanisms (GO:0009757, GO:0010200) (**Figure 10H**).

KEGG gene in the yellow module of ZZ9R seedlings was enriched to ko05120, ko00670, ko00720, ko04966, ko04260, ko05110, ko00523, ko00521, ko05323, ko04391, ko01523, ko00515, ko04390, ko00901, and ko00450 (**Figure 10P**).

**Figure 11:**
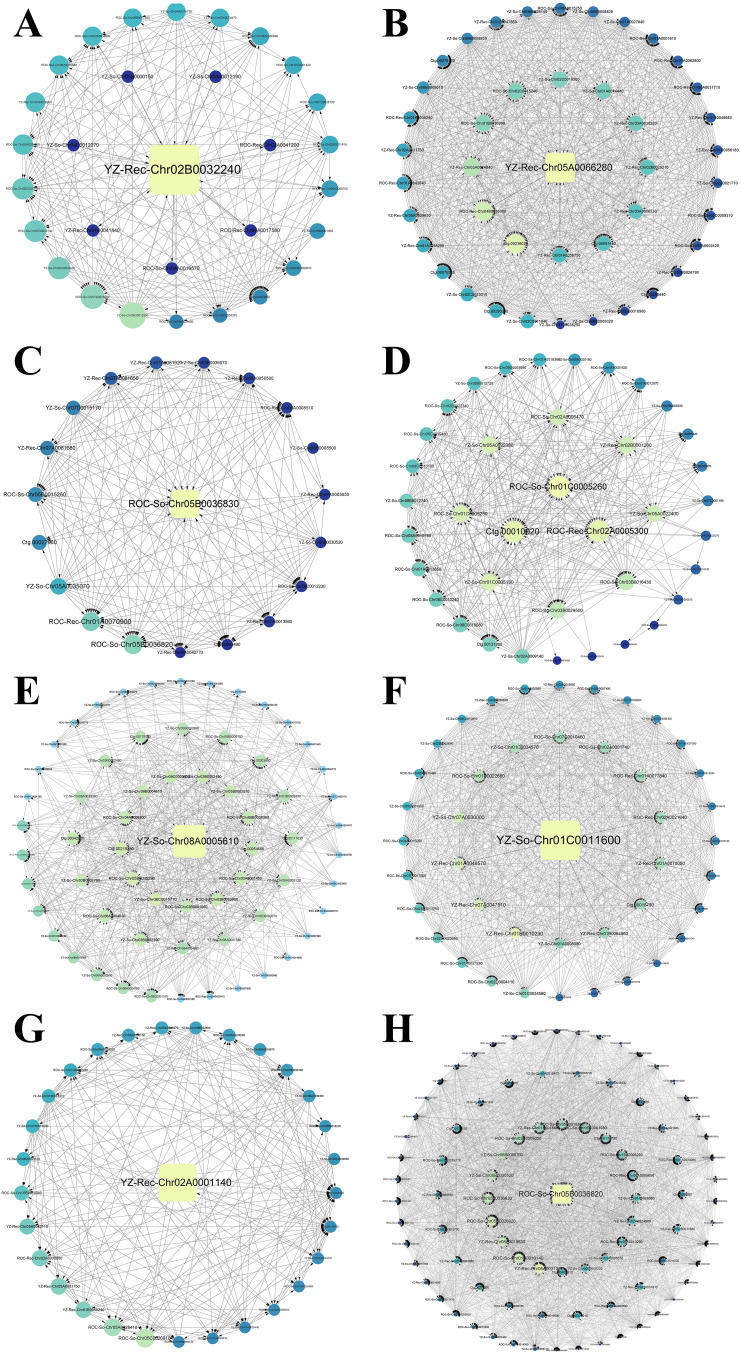
Gene coexpression interaction network of WGCNA module in bud and root tissues of ZZ2 and ZZ9. **(A)** Gene co-expression network for the salmon module in ZZ2B. **(B)** Gene co-expression network for the yellow module in ZZ2B. **(C)** Gene co-expression network for the brown module in ZZ2R. **(D)** Gene co-expression network for the skyblue module in ZZ2R. **(E)** Gene co-expression network for the black module in ZZ9B. **(F)** Gene co-expression network for the tan module in ZZ9B. **(G)** Gene co-expression network for the blue module in ZZ9R. **(H)** Gene co-expression network for the yellow module in ZZ9R.

Genes from the salmon and yellow modules in ZZ2B, the brown and skyblue modules in ZZ2R, the black and tan modules in ZZ9B, and the blue, brown, and yellow modules in ZZ9R were selected based on a threshold criterion of GS and MM > 0.80. These genes were imported into Cytoscape (v3.9.1) for visualizing of co-expression interaction networks, with a focus on highlighting the hub genes with the highest connectivity within each module (**Figure 11**).

The gene with the highest connectivity in the salmon module of ZZ2B is *YZ-Rec-Chr02B0032240*, which is associated with the P21-Rho-binding domain (PBD) (**Figure 11A**). In the yellow module, the most highly connected gene is *YZ-Rec-Chr05A0066280*, which belongs to the tetraspanin family (TM4SF) (**Figure 11B**).

The gene with the highest connectivity in the brown module of ZZ2R is *ROC-So-Chr05B0036830*, which encodes a Chitinase class I (Glyco_hydro_19) protein (**Figure 11C**). In the skyblue module, the most highly connected genes are *ROC-So-Chr01C0005260*, *Ctg.00010020*, and *ROC-Rec-Chr02A0005300*, all of which are associated with the zinc finger (zf-C2H2_6) domain (**Figure 11D**).

The gene with the highest connectivity in the black module of ZZ9B is *YZ-So-Chr08A0005610*, which is associated with O-methyltransferase activity (Methyltransf_3) (**Figure 11E**). In the tan module, the most highly connected gene is *YZ-So-Chr01C0011600*, which is related to the transcription factor (NAM) (**Figure 11F**).

The gene with the highest connectivity in the blue module of ZZ9R is *YZ-Rec-Chr02A0001140*, which is associated with GDSL esterase lipase (Lipase GDSL) (**Figure 11G**). In the yellow module, the most highly connected gene is *ROC-So-Chr05B0036820*, which is related to Chitinase class I (**Figure 11H**).

### 3.7 qRT-PCR validation of transcriptome data and target gene expression

**Figure 12:**
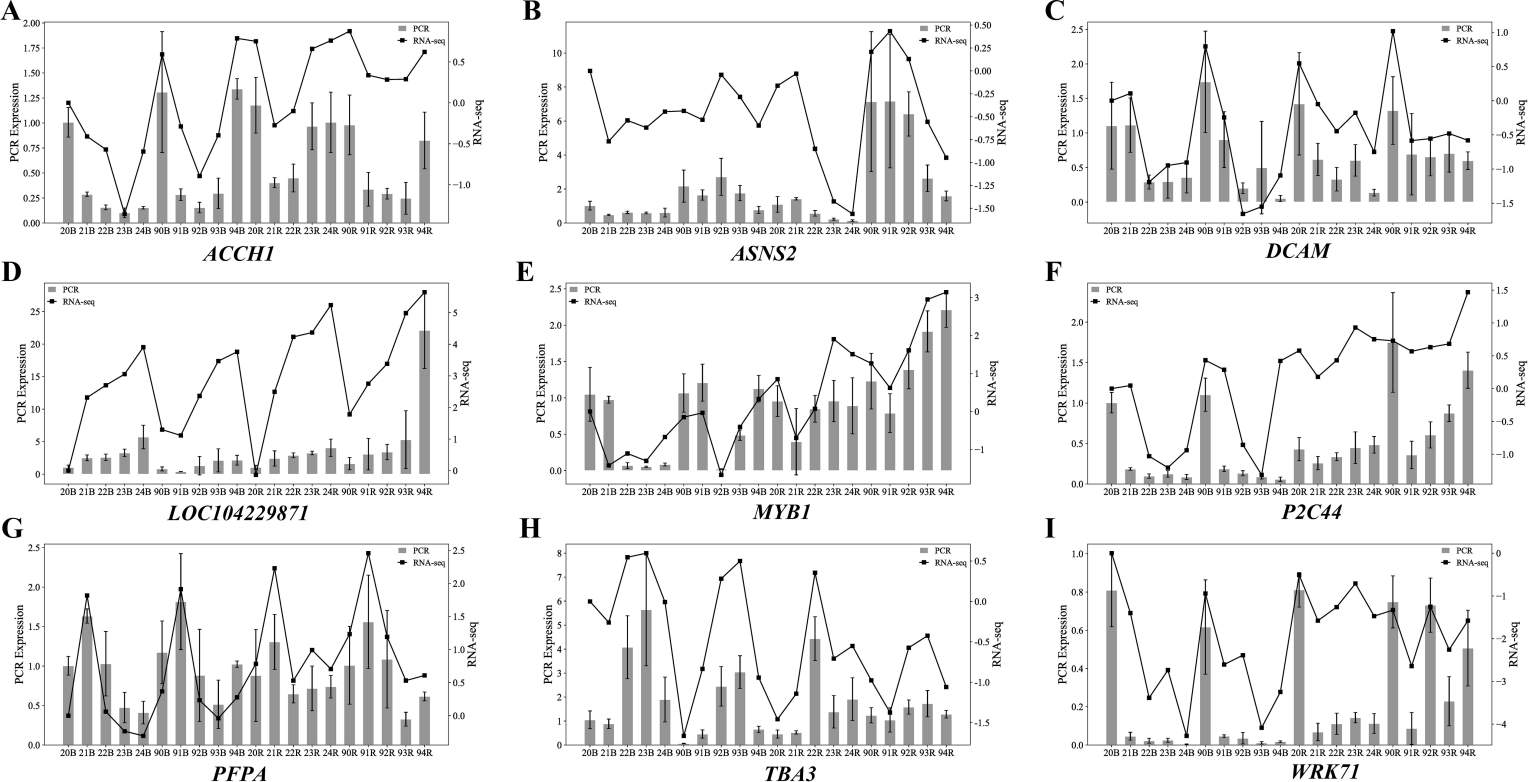
qRT-PCR validation of the representative DEGs identified from the transcriptome analysis. **(A)** qRT-PCR Expression Level Results of *ACCH1*. **(B)** qRT-PCR Expression Level Results of *ASNS2*. **(C)** qRT-PCR Expression Level Results of *DCAM*. **(D)** qRT-PCR Expression Level Results of *LOC104229871*. **(E)** qRT-PCR Expression Level Results of *MTB1*. **(F)** qRT-PCR Expression Level Results of *P2C44*. **(G)** qRT-PCR Expression Level Results of *PFPA*. **(H)** qRT-PCR Expression Level Results of *TBA3*. **(I)** qRT-PCR Expression Level Results of *WRK71*.

Based on the results from WGCNA and differential expression analysis, this experiment selected nine key genes that were potentially associated with sprout development in sugarcane stem for qRT-PCR validation of transcriptome data. *GAPDH* was used as the internal reference gene, and relative expression levels of the nine genes were calculated using the2^-△△Ct^ method. The qRT-PCR results showed trends consistent with the transcriptome data (**Figure 12**), which indicated the reliability of the transcriptomic analysis for sprout development in the stem of ZZ2 and ZZ9.

## 4 Conclusion

We observed that two sugarcane varieties, derived from the same genetic background, exhibit heterochronic development during sprout emergence. ZZ2B sprouts earlier, while ZZ9R emerges sooner. In the cross-sectional analysis of paraffin-embedded sett roots on day 12, ZZ9 exhibited a significantly lower root cortex thickness ratio and a higher vascular cylinder thickness ratio compared to ZZ2, indicating superior water absorption capacity in ZZ9. Through the detection of soluble substances and UHPLC-MS/MS analysis, we identified significant differences in the contents of soluble sugars, IBA, IPA, cZ, ABA, GA_3_, GA_7_, JA, and JA-Ile between the two varieties, which are critical for determining different sprouting strategies for sugarcane setts. Additionally, transcriptomic analysis revealed that the negative regulation of ZZ2’s response to external stimuli is key for the prioritization of axillary bud sprouting, while the Tat complex, a dipeptide transporter, plays a significant role in the preferential development of ZZ9R. WGCNA analysis further demonstrated that the specific metabolism of seed coat mucilage determines the heterochronic development of sett roots and axillary buds.

## 5 Discussion

### 5.1 Effects of dynamic regulation of soluble substances on sprout development in sugarcane setts

Sugarcane setts contain reserves of various storage compounds, primarily in the form of soluble sugars and soluble proteins. These reserves provide essential energy and serve as key prerequisites for plant growth (Sreenivasulu, 2017). Soluble sugars is to supply sufficient nutrients, which further aid in the construction of macromolecules and energy necessary for specific, coordinated development (Sami et al., 2019). During sprouting and root formation, catabolic processes occur, which supply energy and structural materials essential for the growth and development of setts and root systems (Ren et al., 2023). More materials and energy can be provided when the soluble sugar content is higher. Therefore, a strong positive correlation exists between sprout and root development and soluble sugar content (Zhou et al., 2020).

ZZ2 exhibits faster sprouting, which lead to a rapid depletion of stored soluble sugars. This phenomenon causes their content to decrease quickly. By contrast, ZZ9 shows a delayed sprouting, with the consumption of soluble sugars slower than their conversion rate. As a result, the soluble sugar content continues to rise by the fourth day. Similarly, ZZ9R experience earlier root emergence. By the third day of cultivation, a significant increase in root numbers is observed. Correspondingly, a rapid decline in soluble sugar content in the roots is found on the third day. On the contrary, ZZ2 has fewer roots and slower root elongation, which result in lower consumption of soluble sugars. Thus, the soluble sugar content increases as dormancy is broken. In general, soluble sugars are rapidly consumed in the regions of the sugarcane stem nodes where development is prioritized, leading to a decrease in their concentration. Conversely, in the slower-developing regions, the conversion of starch into soluble sugars results in an accumulation of sugars. The sprouting and elongation of axillary buds and sett roots in sugarcane, along with their consumption and conversion of soluble sugars, indicate that changes in soluble sugar content reflect distinct patterns of sugarcane seedling sprouting. In addition, sugars, which function as nutrients and signaling molecules, promote cell division and differentiation in plants. Exogenous application of low concentrations of sugar can enhance seed germination, while high concentrations of sugar inhibit germination in a concentration-dependent manner (Sami et al., 2016). The soluble sugar content in ZZ2B, prior to cultivation, may be within a concentration range that promotes the sprouting of stems. In ZZ9B, soluble sugar levels only reach parity with the initial levels in ZZ2 after two days of cultivation, which mark the entry into the bud scale shedding stage associated with sprouting (Verma et al., 2013).

The trends in soluble protein and soluble sugar levels in ZZ2 and ZZ9 are similar during sprouting. In ZZ2B, soluble proteins are gradually mobilized from their stored state, which results an increase in their levels. Meanwhile, the consumption of soluble proteins during sprouting leads to a decrease in their concentration. The rate of soluble protein synthesis in the stem matches the rate of metabolic degradation, which maintains a constant total level to sustain dynamic equilibrium. In ZZ9B, only bud scale shedding occurs without subsequent bud elongation; therefore, soluble protein levels in the axillary buds continue to accumulate and show an upward trend. Notably, although the initial soluble protein levels differ significantly between axillary buds and sett roots, this difference does not appear to be a determining factor for the preferential sprouting or rooting in sugarcane. Instead, changes in soluble protein levels appear to be dynamically regulated in synchrony with either sprouting or rooting processes.

ZZ9 has opted to develop and elongate its root system, which enable the initial absorption of external nutrients and water. This condition allows it to enter an autotrophic mode. It accumulates energy and nutrients until it can meet the energy demands required for sprouting and continued root growth. By contrast, ZZ2 prioritizes sprouting over root development, which keeps it in a passive state of high dependency on the stem for energy and nutrients while maintaining a heterotrophic mode. Under these conditions, the growth and development of the bud, roots and other organs compete for the energy and materials they need. The limited resources in the stem are insufficient to support all these physiological activities, which may explain why ZZ2, with its sprouting-first strategy, lacks the agricultural advantages observed in ZZ9. In summary, the variations in soluble sugar content in the axillary buds of the stem and in the sett roots between different varieties act as an overarching factor influencing their choice of sprouting or rooting timing. Therefore, the sprouting strategy can be preliminarily assessed by examining the soluble sugar content differences across various tissues within the stem. This approach could help in selecting target sugarcane varieties optimized for agricultural production.

### 5.2 Sett roots of ZZ9 exhibit enhanced water absorption capacity

Root system architecture (RSA) significantly influences plant fitness, crop performance, and grain yield; however, this role has only recently gained appreciation (Rogers and Benfey, 2015). The differing sprouting times of ZZ2R and ZZ9R reveal clear structural differences in their root systems: the cortex-to-stele area ratio in ZZ2 is greater than in ZZ9, whereas the vascular-to-root area ratio in ZZ2 is less than that in ZZ9. After water is absorbed by the root system, it first moves radially from the soil into the roots. It passes through multiple concentric layers before entering the xylem, where it is transported axially to the stem. The absorption of water by the roots is primarily influenced by radial and axial resistances (Kalra et al., 2024). A relatively lower cortex-to-stele area ratio can reduce radial resistance to water absorption, while a relatively higher vascular-to-root area ratio ensures adequate longitudinal water transport capacity. This difference in root structure suggests that ZZ9 has a greater capacity for water absorption and transport than ZZ2. This observation is also consistent with field experiments, which showed that ZZ9 exhibits stronger drought resistance (Yin et al., 2020). In addition paraffin section analysis of the two varieties reveals that ZZ9R possess more cavity structures (Zhang et al., 2023). This prioritization of root emergence in ZZ9 may facilitate quicker progression to maturity. This condition underscores the earlier root development observed in ZZ9 than in ZZ2.

### 5.3 Negative regulation of external stimuli response promotes preferential sprouting in ZZ2

The sprouting of sugarcane axillary buds is a complex physiological process that involves multiple biological processes and molecular mechanisms, including environmental stress response, DNA replication, energy metabolism, nutrient signaling, and redox signaling. Negative regulation of the defense response to external stimuli may be a key factor promoting axillary bud sprouting. Overall, the differentially upregulated genes are significantly enriched in pathways related to DNA replication and metabolism in plant plastids and mitochondria (GO:0033258, GO:0033259, GO:0006264, GO:0032042, GO:0000002). Plastids are double-membraned organelles that are primarily found in plant cells and certain algal cells, including chloroplasts, chromoplasts, leucoplasts, elaioplasts, and amyloplasts. They are mainly responsible for photosynthesis, pigment synthesis, storage of various substances, and metabolic activities (Kuntz et al., 2024). Active DNA replication and metabolism in the plastids and mitochondria of ZZ2 suggest that cell division, differentiation, energy conversion, and storage are highly active in its axillary buds. Furthermore, the enrichment of these pathways indicates that ZZ2B are well-prepared for sprouting, particularly in terms of cell division, differentiation, and energy utilization.

The significant enrichment of the linoleic acid metabolism pathway (ko00591) suggests extensive synthesis of lipid peroxides in ZZ2B (Yan et al., 2021). GO analysis also identified enrichment in several linoleic acid metabolism-related terms (GO:1990136, GO:0016165, GO:0016702, GO:0016701), which further support the validity of the enrichment analysis results. Lipoxygenases, which are a class of oxidoreductases, are confirmed to play a role in seedling development and primarily catalyze the oxygenation of linoleic or linolenic acids (Vick and Zimmerman, 1981). The role of lipoxygenases in early plant development remains unclear. Studies indicate that the oxygenation of lipids occurs prior to their catabolic breakdown, with hydroxy-octadecadienoic acid—a degradation product—acting as an endogenous substrate for β-oxidation. Consequently, it acts as a carbon source for growing barley embryos (Holtman et al., 1997). In the storage state, lipids serve as the primary carbon source for seedlings. Mobilization of these stored lipids is a key feature of plant germination (Feussner et al., 1995). Thus, the significant enrichment of the linoleic acid metabolism pathway suggests active breakdown and metabolism of stored lipids during the sprouting process of ZZ2, which ensure a sufficient carbon supply to support its growth. This process is crucial for the sprouting of sugarcane axillary buds.

Notably, the plasmid-induced replication and metabolism of mitochondrial DNA of ZZ2, along with linoleic acid metabolism, may be a result of negative regulation in response to external environmental stimuli (GO:1900366, GO:2000068, GO:0002213, GO:0002832, GO:0032102). This phenomenon could trigger a series of chain reactions, which weaken the defense mechanisms of the plant under complex regulatory processes to allocate energy storage materials. Accordingly, the sprouting and growth of axillary buds are activated. Ultimately, earlier sprouting of ZZ2 occurs.

### 5.4 Twin-arginine transporter complex promotes early development of the sett roots of ZZ9

The differentially upregulated genes in ZZ9R are specifically enriched in pathways related to the Tat complex, which indicates the important role of Tat in the sprouting of sugarcane sett roots. Tat is a protein transport system found in the thylakoid membranes of plants, the inner membranes of certain bacteria, and the mitochondrial membranes of some organisms. This complex is essential for translocating folded proteins across membranes, particularly those proteins with a twin-arginine (RR) motif in their signal peptides (Robinson and Bolhuis, 2004). The Tat complex typically consists of three essential proteins: TatA, TatB, and TatC. In the presence of a substrate, the Tat complex assembles on the membrane. The substrate protein is then directed to the TatBC subcomplex, where it remains bound in an unfolded or partially folded state. Upon binding, the TatC component facilitates the movement of the substrate protein toward the TatA channel. TatA oligomerizes to form a transport channel, which allows the folded protein to pass through the membrane (Yen et al., 2002; New et al., 2018). This unique characteristic distinguishes the Tat system from other transport systems, such as the Sec pathway, given that the latter typically transports unfolded proteins. The Tat transport process is dependent on the proton motive force (PMF) across the membrane, which provides the energy to drive folded proteins through the TatA channel. Proteins transported by the Tat complex often play critical roles in photosynthesis and respiration, including components of the electron transport chain and enzymes involved in metabolic pathways (Robinson and Bolhuis, 2001). Unlike other protein transport systems, the Tat system can transport already folded proteins. Many key proteins involved in photosynthesis and metabolism within the chloroplast are transported via the Tat pathway, such as components of the thylakoid membrane that are involved in Photosystem I, Photosystem II, the cytochrome b6f complex, and ATP synthase. These proteins are crucial for maintaining chloroplast function and the efficiency of plant photosynthesis (Robinson et al., 2001). During seed development, carbon is reallocated from maternal tissues to support sprouting and subsequent growth. As this pool of resources is depleted post-sprouting, the plant begins autotrophic growth through leaf photosynthesis. Photoassimilates derived from the leaf sustain the plant and form new organs, including other vegetative leaves, stems, bracts, flowers, fruits, and seeds. In contrast to the belief that reproductive tissues act only as resource sinks, many studies demonstrate that flowers, fruits, and seeds are photosynthetically active (Brazel and Ó Maoiléidigh, 2019). Research indicates that chloroplasts play an important role in root development and sprouting (Kobayashi et al., 2013; Kang et al., 2014; Li et al., 2021). The active expression of the Tat system suggests that functions performed by various plant plastids, such as chloroplasts, may promote root sprouting in sugarcane seedlings. Exploring the potential correlation between the Tat system and the root sprouting phenotype could be an intriguing direction for future research.

### 5.5 Metabolism of seed coat mucilage: a key factor in sprouting and root formation consuming valuable rhizome resources

We found a significant correlation between GAs and ZZ2B, as well as between CKs and ZZ9R seedlings. In addition, PLS-DA analysis indicated that GAs and CKs are differential metabolites. Thus, we focus on their roles in determining sprouting and root formation.

GAs promotes a range of developmental and growth processes in plants, especially sprouting, elongation growth and flowering time (Schwechheimer and Willige, 2009). GAs are a large class of tetracyclic diterpenoid compounds, and approximately 136 forms have been identified in higher plants and fungi. However, only a few of them, including GA_1_, GA_3_, GA_4_, and GA_7_, are biologically active, while other GAs are intermediate forms in the GA biosynthetic process or inactive forms of Gas (Shah et al., 2023).

GA signaling is another key step controlling the transcription of GA-dependent genes and the regulation of the GA response. Similar to other plant hormones such as JA, auxin, and strigolactone, the GA signaling process is based on E3 ubiquitin ligase-mediated proteolysis of DELLAs (Jang et al., 2020).

In this study, gibberellins broadly influenced the physiological processes of axillary buds in sugarcane stems, primarily by controlling energy and sugar metabolism-related processes through the negative regulation of the gibberellic acid-mediated signaling pathway (GO:0009938). The influence included pathways such as glycolysis (GO:0006096) and pyruvate production (GO:0042866), which supply ATP to axillary buds to support rapid cell growth for prioritizing bud sprouting.

Interestingly, enrichment results indicate that gibberellins regulated 67 genes in the jasmonic acid-mediated signaling pathway (GO:2000022) in ZZ2B. In ZZ9R, cytokinins regulated 36 genes involved in the jasmonic acid biosynthetic process (GO:0009695). This result suggests a synergistic regulation of sugarcane sprouting and root formation by GAs and JAs. Many studies reported that developmental flexibility under stress conditions largely depends on the interplay between stress-related hormones and growth-related hormones, with increasing evidence indicating that JA and GA antagonistically interact to coordinate plant growth and defense (Pan et al., 2023). Extensive research on the JAZ-JA signaling repressor proteins, and the DELLA GA signaling repressor proteins reveals that direct interaction between JAZs and DELLAs mediates the antagonistic interaction between JA and GA. In the “relief of repression” model, the JAZ-DELLA interaction attenuates the functions of JAZs and DELLAs as signaling repressors. For example, in GA-free conditions, DELLAs directly interact with JAZs and allow MYC2 to promote the JA response. When GA is present, JAZs are released from the DELLA-JAZ complex by degradation of DELLAs. The free JAZs then attenuate the JA response by directly interacting with MYC2. This model illustrates the DELLA-mediated upregulation of the JA response and the antagonistic interaction between JA and GA. Supporting studies show that JA promotes the transcription of *RGA3*, with the JA-responsive MYC2 transcription factor directly binding to the promoter of *RGA3* (Jang et al., 2020).

We found that under the co-regulation of various hormones, such as GAs, CKs, and JAs, the metabolic pathways of mucilage biosynthetic process involved in seed coat development (GO:0048354, GO:0048359) and glucuronoxylan biosynthetic process (GO:0010417, GO:0010413, GO:0045492, GO:0045491) were significantly enriched in ZZ2B and ZZ9R.

Seed coat mucilage primarily consists of polysaccharides that cover the outer layer of the seed, which promotes seed hydration and germination. This process enhances seedling emergence rates and reduces seedling mortality. Four types of polysaccharides have been identified in the mucilage, including xylan, pectin, glucomannan, and cellulose (Tsai et al., 2021). Although no current studies have indicated whether the sett stems of sugarcane possess seed coat mucilage, the results of this enrichment analysis suggest that seed coat mucilage also plays an important role in the sprouting process of sugarcane stems. Research indicates that seed coat mucilage plays a crucial role in promoting seed germination under adverse conditions. Identifying key genes involved in the biosynthesis and release of seed coat mucilage can provide potential targets for cultivating stress-resistant crops, which improves agricultural productivity and environmental resilience (Ma et al., 2024). This novel approach and direction for agricultural production in sugarcane is noteworthy. The production of seed coat mucilage imposes a considerable metabolic cost on plants (Liu et al., 2021), particularly in terms of soluble sugar consumption. When ZZ2B sprouts, producing seed coat mucilage consumes a significant portion of the nutrient resources and energy from the stem. Although this process prioritizes the sprouting of ZZ2, it subsequently imposes greater challenges on ZZ2 during later stages of growth and development due to a lack of sufficient foundational material support. Therefore, GAs, CKs, and JAs mediate the metabolic differences of seed coat mucilage across various tissues, which results in distinct sprouting strategies for sprouting versus rooting. This differential resource allocation within the stem ultimately contributes to the markedly different agronomic traits observed between the two sugarcane varieties.

## Data Availability

The Reference genome data for transcriptome presented in the study are deposited in the National Genomics Data Center repository, accession number: GWHEQVP00000000. Transcriptome data reported in this paper has been deposited in Sequence Read Archive (SRA) repository, BioProject: PRJNA1207606.
