# Supplementary figures and images for: Identification of functional pathways and hub genes associated with the heterochronic development of sugarcane axillary buds and sett roots through multi-omics analysis
Source: Front Plant Sci. 2025 Mar 14;16:1551783. doi: 10.3389/fpls.2025.1551783 (PMC11949976; doi:10.3389/fpls.2025.1551783)

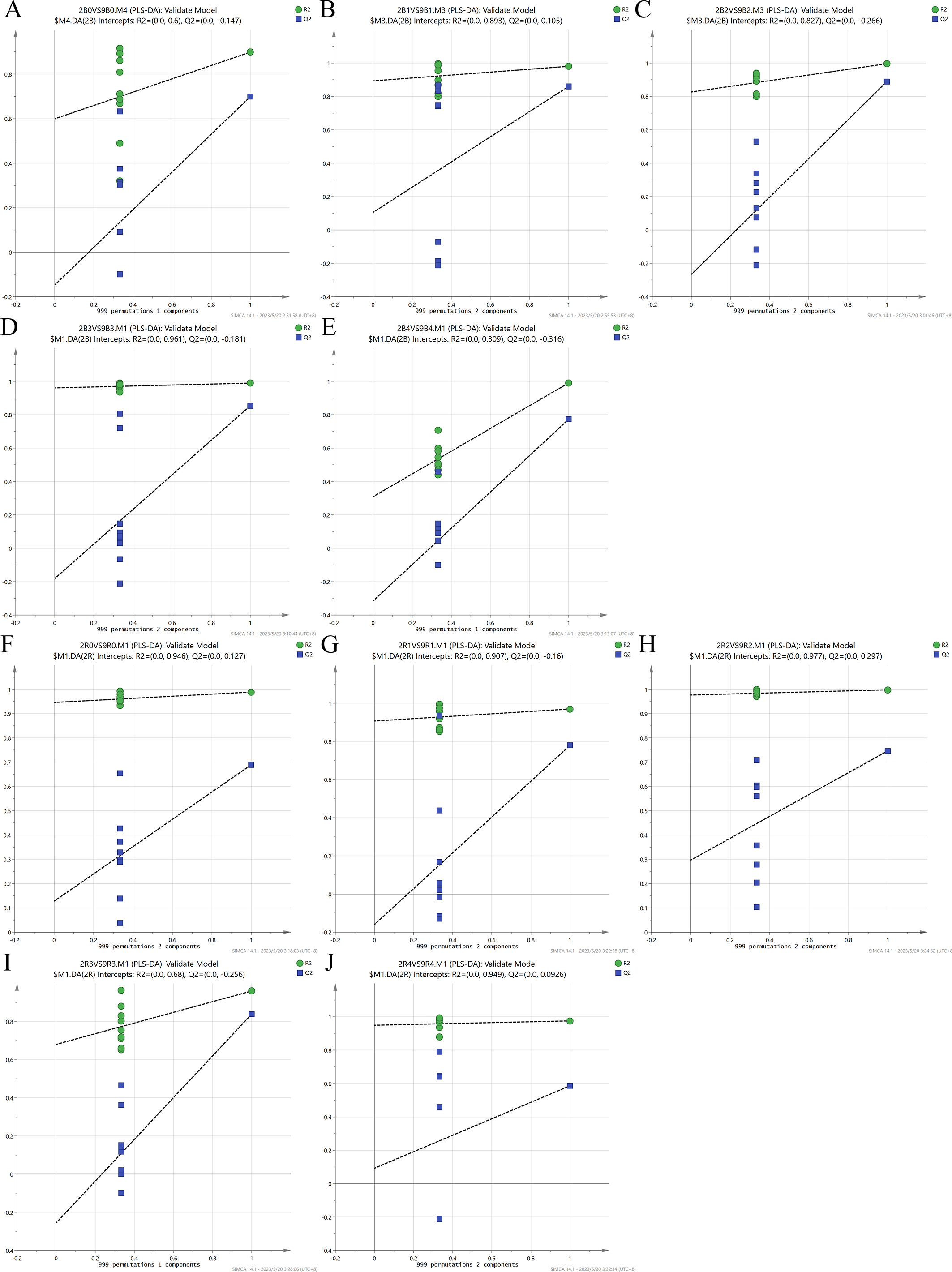

Supplement: Supplementary file 1 [file Image1.jpeg]

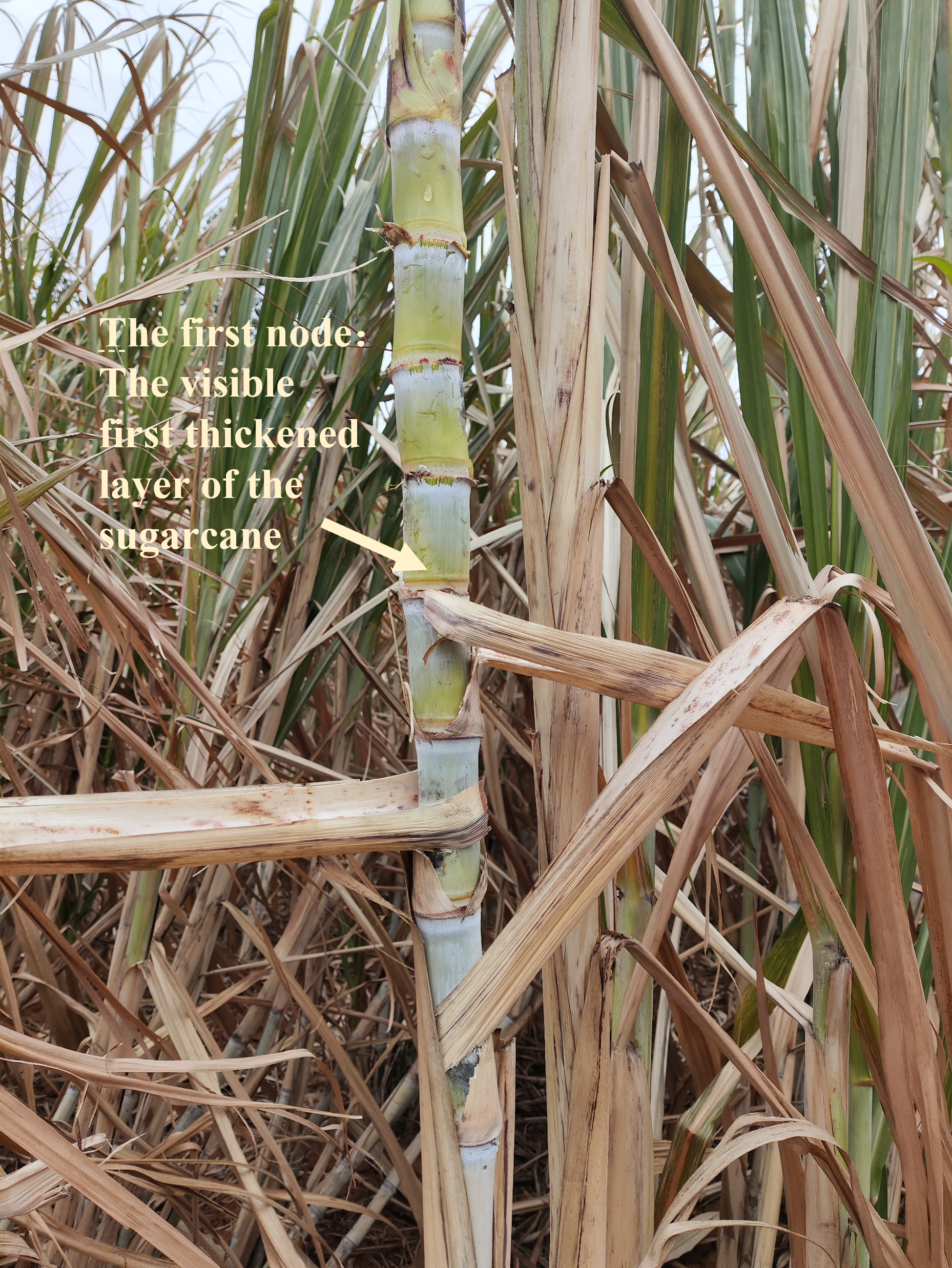

Supplement: Supplementary file 3 [file Image3.png]

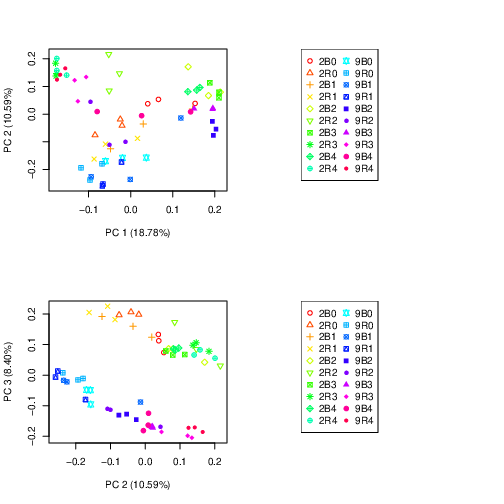

Supplement: Supplementary file 4 [file Image4.png]
